# ‘That Essence of Empowerment’: An Exploration of People Living With Mental Illness', Healthcare Professionals', and Researchers' Experiences of Co‐Design

**DOI:** 10.1111/hex.70888

**Published:** 2026-10-15

**Authors:** Catherine O'Donnell, Oliver Ardill‐Young, Jacqueline Huber, Aaron Schokman, Philip B. Ward, Jackie Curtis, Alyssa Milton, Scott Teasdale

**Affiliations:** ^1^ Mindgardens Neuroscience Network Randwick New South Wales Australia; ^2^ Discipline of Psychiatry and Mental Health, School of Clinical Medicine, Faculty of Medicine University of New South Wales Kensington New South Wales Australia; ^3^ South Eastern Sydney Local Health District Sydney New South Wales Australia; ^4^ St Vincent's Hospital, Darlinghurst Sydney New South Wales Australia; ^5^ Sydney School of Medicine (Central Clinical School) The University of Sydney Sydney New South Wales Australia; ^6^ Australian Research Council (ARC) Centre of Excellence for Children and Families over the Life Course Sydney New South Wales Australia; ^7^ Schizophrenia Research Unit Ingham Institute for Applied Medical Research Liverpool New South Wales Australia; ^8^ Brain and Mind Centre The University of Sydney Sydney New South Wales Australia

**Keywords:** co‐design, health equity, mental health, psychiatry, qualitative research

## Abstract

**Aims:**

Co‐design is commonly adopted as a methodology within mental health research. However, little is known about the experiences of people involved in co‐design. We aimed to describe how a recent co‐design process was enacted, exploring experiences of this and how power was manifested through this process.

**Methods:**

Two workshops, seven focus groups, and a structured power reflection were conducted as part of an iterative co‐design process involving people living with mental illness experiencing food insecurity (*n* = 9), mental healthcare professionals, including peer workers (*n* = 11), and researchers (*n* = 4). These took place in Sydney, Australia, between March 2023 and February 2024. Experiences of co‐design were explored using a mixed‐methods survey following workshops and qualitative enquiry during focus groups.

**Results:**

Three analytic categories were constructed, relating to ‘doing’ co‐design, the experience of co‐design and the impacts of co‐design, comprising 12 interpretative themes. The development of strong relationships, through the diversity of collaboration and open communication, was perceived as contributing to the impact of overall positive experiences and personal growth. Challenges centred around managing expectations of the uncertain process and perceptions regarding power imbalances. Co‐design was also seen as having flow‐on effects on clinical culture and snowballing action within the health service.

**Conclusions:**

We identified mechanisms of how co‐design processes relate to experiences and impacts. Inequalities in power dynamics are inescapable but can be successfully navigated by building a sense of partnership and collaboration. Crucially, our findings emphasised the intrinsic value of including people with Lived/Living Experience of mental illness in research which directly impacts them.

**Lived Experience or Public Contribution:**

Two co‐authors with Lived/Living Experience contributed directly to this article, one of whom is the first co‐author and co‐led the research from initial conceptualisation and funding acquisition. Findings were shared with all participants, as well as with a wider group of Lived/Living Experience representatives and healthcare professionals, who informed interpretation and dissemination.

## 1 Introduction

Throughout history, people with Lived/Living Experience (PWLE) of mental illness have often been excluded from research and decision‐making about their own lives [1, 2]. This exclusion within systems which provide care has frequently led to a legacy of disempowerment and mistrust [3]. As a response, mental health services globally are beginning to embed into policy the need for engagement with PWLE in their design, implementation, and evaluation—though this varies dramatically in its practical realisation across contexts [4, 5, 6, 7, 8, 9, 10]. This is occurring as part of a wider movement within the health sciences, democratising how knowledge is produced and recognising expertise through experience, to encompass a fuller spectrum of approaches to understanding evidence.

The past decade has seen a rapid growth in participatory research methods, termed by some as the ‘Participatory Zeitgeist’ [11]. However, the ways in which engagement with PWLE can occur vary greatly across studies. Many names are used to refer to these methods, including (but not limited to) co‐design, public and patient involvement and engagement, co‐production, participatory action research, and user‐led research. These approaches are often conceptualised as existing on a continuum frequently illustrated using Arnstein's ladder of participation [12], although different levels may be more appropriate at different stages of research. However, these terms are often used interchangeably despite important conceptual differences. For example, co‐design, the approach focused on in this article, is outlined by Bellingham et al. [13] as research possessing the qualities outlined in Box 1. In practice, however, the term may be used to refer to partnership across all stages of the research process or to more consultative engagement. This variability creates challenges for interpretation, evaluation, and comparison across studies.

Box 1 **Co‐design qualities of research participation.**

**Table:** 

LE and/or Peer researchers have experience relevant to the area of the research study, and work in partnership with conventional researchers in all stages (substantive) or key stages (bare‐minimum) of the research process. Decision making is shared and equitable, and decisions related to, or that impact, the codesigned elements of the project are made together. Number of LE/Peer researchers and conventional researchers are at least equal, or LE/Peer researchers form the majority. LE/Peer researcher perspectives are privileged, power differentials are addressed. Expertise is equally valued, but LE/peer perspectives are privileged in discussions.

With these policy changes and increasing stipulation by funding bodies for inclusion of PWLE in research design, efforts to evaluate the impact of participation have increased. This is aligned with an epistemic justification for participation, seeing it as a way to improve the ‘three Rs’: the rigour of research, its relevance to the community, and its reach [14]. Evidence supporting the benefits of these three aspects has been reported across the broader health sciences and within mental health, and a large body of research examining the impacts of participatory approaches. However, in their meta‐review of co‐design in the health sciences, Slattery et al. [15] note that co‐design ‘appears to be widely used but seldom described or evaluated in detail’. Similarly, two recent scoping reviews [16, 17] of the engagement of PWLE, specifically within substance use and mental health research, found a range of positive impacts on the research process. Yet, both reported inconsistency in detailing exactly how PWLE were engaged in the research process and how this contributed to the impacts of the research.

Sheikhan et al. [16] also report that most studies in their review focus on the epistemic benefits of Lived/Living Experience (LE) inclusion, while giving less attention to an ethical imperative: that those directly impacted by research have a legitimate claim to contribute meaningfully to it. The ethical and epistemic justifications are interwoven and together underpin participatory approaches. However, Friesen et al. [18] highlight the potential harms of a focus on impact and lack of a focus on process and experience within mental health research. If anticipated epistemic benefits (e.g., increased participant recruitment or improved intervention relevance) are not realised, this could contribute to scepticism about the value of participatory approaches. When the involvement of LE is required, but researchers may be sceptical or uncertain about how to implement it meaningfully, participation may be tokenistic and perceived as ‘box ticking’. In addition, when LE involvement is framed primarily as a means of improving research outcomes, there is a risk that the experiences and needs of those involved receive less attention. In such circumstances, participation may be experienced as tokenistic or extractive, potentially reinforcing existing power imbalances or contributing to feelings of stigmatisation or disempowerment. However, when participatory processes are implemented with careful attention to relationships, support, and shared decision‐making, they may also foster empowerment, validation, and more equitable research partnerships.

Broadening the scope of evaluation beyond research outputs to consider people's experiences of involvement is therefore necessary. This includes attention not only to the quality of the research produced but also to the quality of participation in co‐design [19, 20, 21]. This has been identified as an evidence gap through a scoping review [22] and validated as a gap and research priority through deliberative and consultative engagement with PWLE, carers, and researchers [23]. In their review, Sheikhan et al. [16] also identified the impact of engagement on individuals as a key focus within the literature, yet note that few studies explicitly interrogate power [24]. Many discuss these experiences only as effects, minimally exploring how they are enacted through participatory processes [16]. While a strength of the review is a synthesis of PWLEs', researchers', and other stakeholders' experiences, studies that examine these perspectives together within the same context remain limited. Greater attention to how participation is enacted in practice, including how influence and decision‐making are shared between participants and researchers, is therefore needed to better understand how power operates within co‐design.

The current paper is part of a larger project exploring mental illness and food insecurity. As part of this project, we undertook a co‐design process to understand how PWLE of severe mental illness and healthcare professionals (HCPs) in Australia perceive the causes, experience, and impacts of food insecurity [25]. The current paper explores the co‐design process, rather than the co‐design of the research process itself. Addressing the issues highlighted above, this paper aimed to: (a) describe in detail the co‐design process undertaken (‘How was co‐design enacted?’), (b) understand PWLEs', HCPs', and researchers' experiences of participating in the same co‐design process (‘How was co‐design experienced?’), and (c) critically reflect on the power dynamics throughout the co‐design process (‘How was power manifested in the co‐design?’). We first describe the co‐design process to provide context, before describing how this process was evaluated.

## 2 Methodology

### 2.1 Research Design and Approach

The design of the co‐design process was principally guided by McKercher's co‐design principles, mindsets and processes [26]. Broadly, reflecting retrospectively, we aligned with a ‘bare minimum’ of co‐design in terms of the research team make‐up [27]: at least one LE researcher was actively involved in all stages of the research cycle, in a team made up of a majority of researchers identifying as ‘conventional’ researchers (i.e., with learned experience) [28]. In terms of the level of participation of PWLE, Supporting Information S1: File 1 maps key aspects using Daya's checklist—completed retrospectively [29].

This paper is a mixed‐methods evaluation of the co‐design process. The evaluation components consisted of analysis of surveys from surveys, focus groups, structured reflection, and field notes. An overview of the research process is provided in Figure 1.

**Figure 1:**
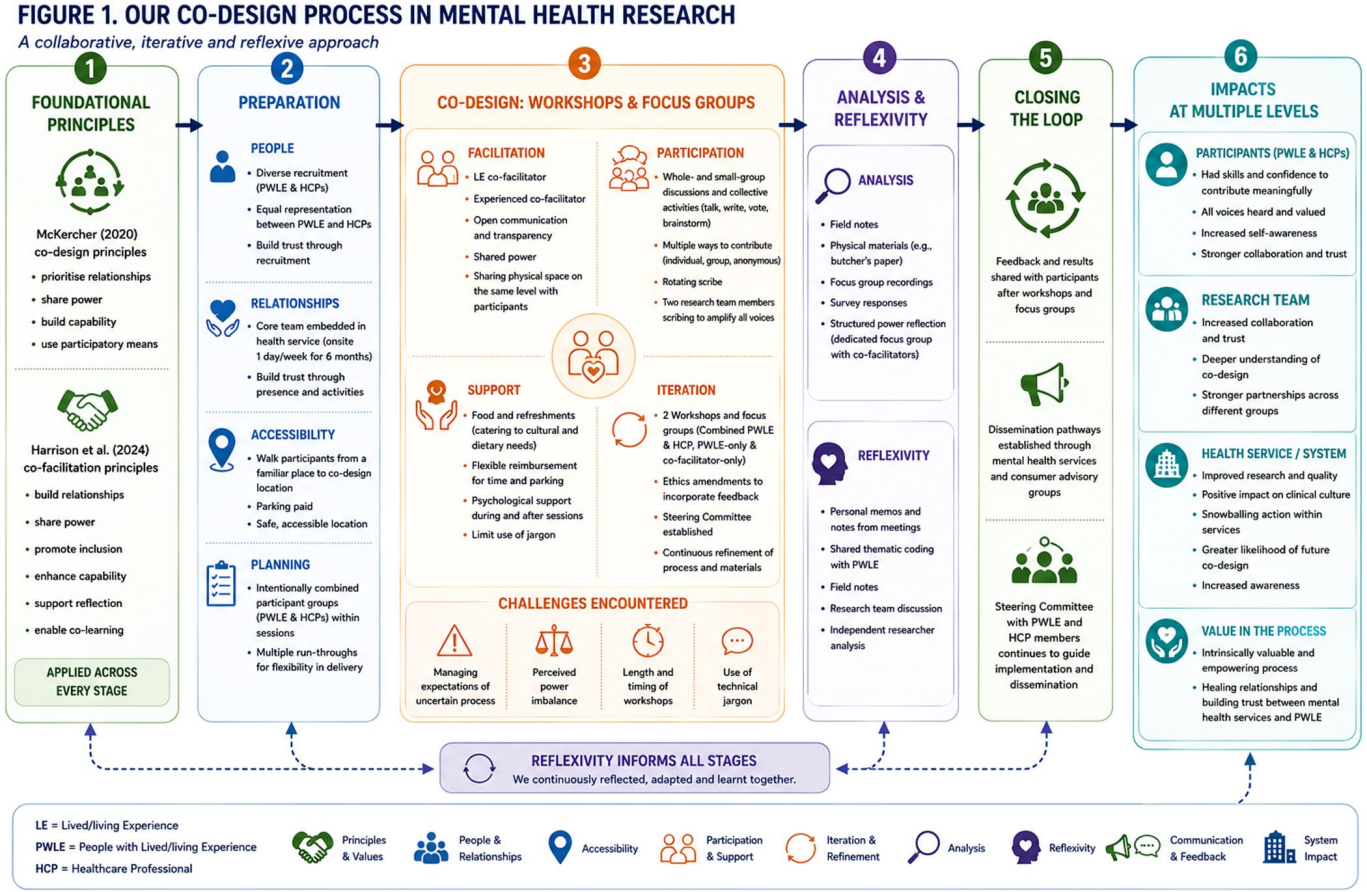
Research process.

This research received ethical approval from the South Eastern Sydney Local Health District (SESLHD) Human Research Ethics Committee (Reference: 2022/ETH02327). Reporting is guided by the GRIPP2‐SF checklist [30] and Reflexive Thematic Analysis Reporting Guidelines [31] (Supporting Information S1: File 2 and Supporting Information S3: File 3).

### 2.2 Co‐Design Process

#### 2.2.1 Setting and Participants

Co‐design was conducted in the metropolitan region of south‐eastern Sydney, Australia, between March 2023 and February 2024. It involved three distinct participant groups: (1) community‐dwelling adults living with severe mental illness engaged with mental health services and able to provide informed consent (determined by their clinical team), who were experiencing moderate to severe food insecurity (assessed through a validated structured interview) [32] and comfortable communicating in English; (2) HCPs from the community mental health service such as nurses, allied health clinicians, or peer workers; and (3) co‐design co‐facilitators. Four co‐facilitators participated in a focus group. In addition, two of these co‐facilitators—the two project co‐leads (*n* = 1 traditional researcher and *n* = 1 LE researcher) involved in research conceptualisation from the outset—also participated in a structured reflection on power dynamics throughout the co‐design process. In total, 15 PWLE expressed interest in participating, and 9 participated over the course of the co‐design process (*n* = 5 attended all sessions). Fifteen HCPs expressed interest in participating, and 11 participated (*n* = 3 attended all sessions). A detailed participant flow for PWLE and HCPs is provided in our earlier study [25]. Table 1 displays demographic details of PWLE and HCPs.

**Table 1:** Demographics of participants.

Variable	PWLE (*N* = 9)		HCPs (*N* = 11)	
	*N*/Mean	%/SD	*N*/Mean	%/SD
Gender				
Male‐identifying	2	22.2%	0	0%
Female‐identifying	4	44.4%	10	90.9%
Non‐binary	2	22.2%	1	9.1%
Prefer not to say	1	11.2%	0	0%
Previous co‐design experience				
Yes	2	22.2%	9	81.8%
No	7	77.8%	2	18.2%
Cultural background^a^				
Australian	6	66.7%	N/R	N/R
New Zealander	2	22.2%		
Aboriginal	1	11.2%		
Maori	1	11.2%		
Irish	1	11.2%		
Spanish	1	11.2%		
Filipino	1	11.2%		
Singaporean	1	11.2%		
Greek	1	11.2%		
Disciplinary background				
Nurse	N/R	N/R	3	41.3%
Social worker			2	18.2%
Occupational therapist			2	18.2%
Peer worker			2	18.2%
Dietitian			2	18.2%

*Note:* N/R – not recorded.

^a^ Cultural background was not recorded as mutually exclusive, but is presented here separately for confidentiality.

#### 2.2.2 Recruitment

PWLE were recruited through expression‐of‐interest sheets provided to them by their case managers at an adult community mental health centre, before a telephone or in‐person meeting with either O. A.‐Y. or S. T. to screen for food insecurity. HCPs were recruited using a ‘maximum variation’ approach regarding disciplinary background and were recruited through a mixture of email and verbal invitations during meetings (e.g., clinical reviews). Those who satisfied the inclusion criteria met with O. A.‐Y. to read through the information and consent form in detail, answering any questions, before taking at least 24 h to consider their decision to provide written consent. Participants provided consent jointly for participating in the co‐design process and participating in the evaluation process. This informed consent procedure was completed for each co‐design session.

Recruitment ceased when pre‐specified target sample sizes for the workshops were reached. We planned to recruit *N* = 10 PWLE and *N* = 8 HCPs based on guidance for workshops and focus groups [33] and to establish equal power dynamics among groups and account for anticipated drop‐out. Participants were invited back for ongoing participation in all co‐design sessions where they continued to meet eligibility criteria. In the case of drop‐out, new participants who had expressed interest were recruited in order to reach the target sample size.

#### 2.2.3 Co‐Design Sessions

Figure 2 provides an overview of the co‐design, including recruitment and preparation, structured to McKercher's processes [26]. Two 6‐h workshops (*N* = 2 with combined PWLE and HCP participants; March and July 2023) and seven 1.5‐h focus groups (*n* = 5 with combined PWLE and HCP participants, *n* = 1 with only PWLE participants, and *n* = 1 with co‐facilitators; October 2023–February 2024) were conducted in total. A 1.5‐h structured reflection on the co‐design process among two co‐facilitators was also conducted in August 2024 [34]. Detailed agendas and question guides of co‐design sessions are available as Supporting Information S4–S8: Files [Link], [Link], [Link], [Link], [Link] and are also described in our previous study from an intervention development perspective [25]. Evaluation surveys are provided as Supporting Information S9: File 9 and Supporting Information S10: File 10.

**Figure 2:**
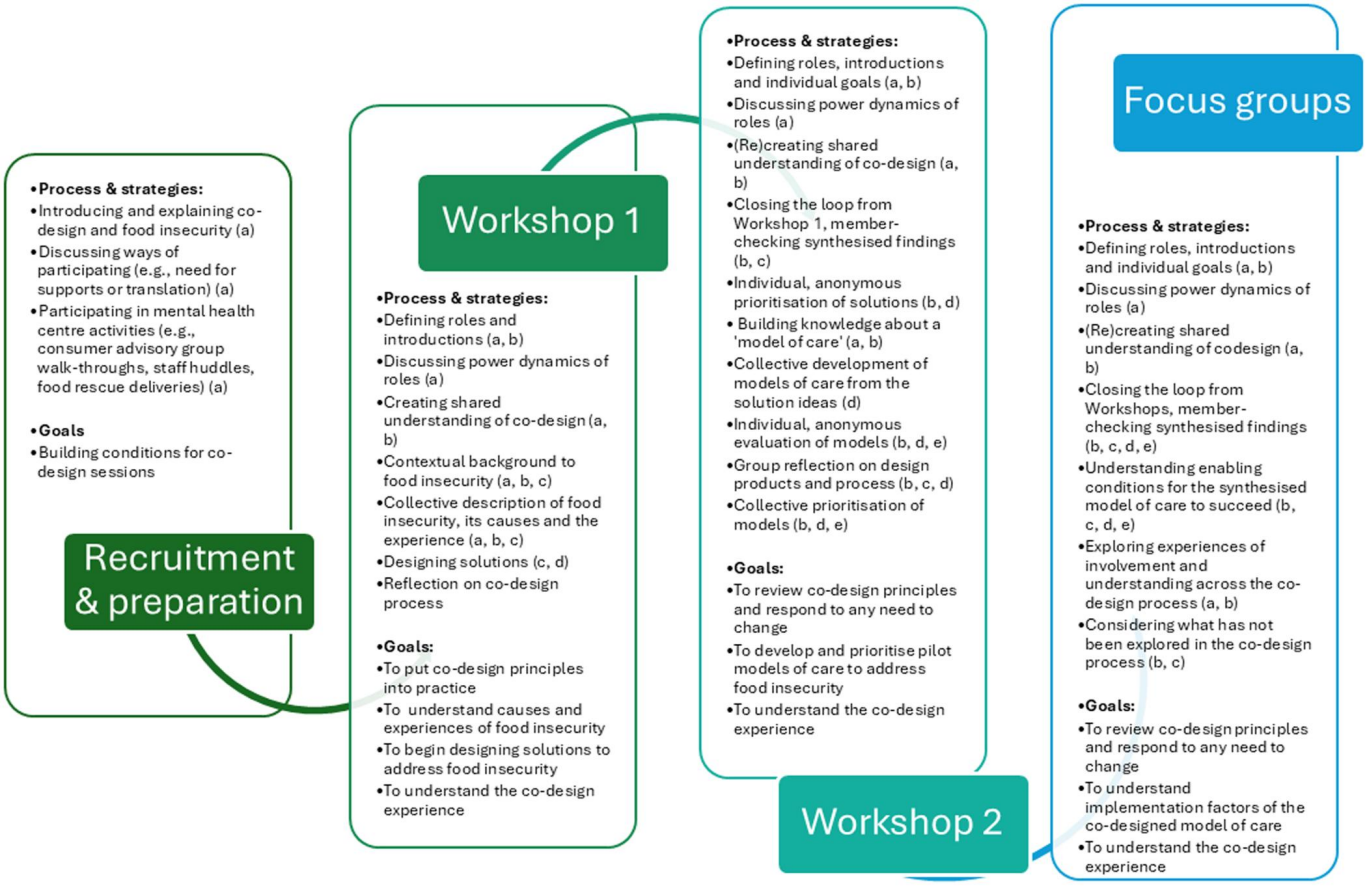
Co‐design sessions. Processes and strategies are marked for alignment with McKercher's [26] process diagram of co‐design: (a) build the conditions, (b) immerse and align, (c) (re)discover, (d) design, (e) test and refine.

McKercher's processes and principles were translated into application at every stage, as illustrated in Figure 2. For example, the beginning of Workshop 2—in defining roles, introductions and individual goals—focussed on the processes of ‘build the conditions’ and ‘immerse and align’. ‘Build the conditions’ is centred on creating the foundations for safe, ethical, and meaningful participation, including clarifying who should be involved, why the work matters, and what conditions are needed for collaboration, while ‘immerse and align’ focuses on developing a shared understanding of the purpose, context, existing knowledge, and collective aspirations for the work. This stage operationalised these process phases by establishing an understanding around power and respect, clarifying roles and expectations, facilitating participant introductions, exploring individual motivations for involvement, and developing a shared understanding of the group's aims and priorities.

This operationalisation was also guided by McKercher's principles [26]: prioritising relationships (by encouraging personal introductions, connection‐building and recognition of participants' experiences and expertise); sharing power (by clarifying roles, valuing all forms of knowledge equally, and creating opportunities for participants to shape the workshop direction); building capability (by orienting participants to the co‐design process, purpose, and expectations); and using participatory means (by utilising facilitated discussion, icebreakers, reflection activities, and collaborative goal‐setting to ensure active involvement from all members).

Co‐design sessions began with Acknowledgements of Country (recognition of the Traditional Custodians of the land) and of LE, icebreakers, and open discussion of roles and power [35]. Workshops were run with HCPs and PWLE, all contributing to the co‐design together, in line with other co‐design research [36]. Importantly, power was introduced from a service user/survivor perspective, and specific power imbalances between HCPs, PWLE, and researchers were explicitly addressed (e.g., reflection on what it means to be powerless, collectively generating strategies to help others feel comfortable) [3, 37]. These actions were designed to build trust and a foundational awareness for sharing power. In addition, co‐design as a way of working together and expectations of what this meant were also collectively discussed and co‐defined.

Workshops combined open discussion as a whole or in small groups of 3–4 people (combining approximately equal ratios of PWLE and HCPs), as well as collective activities (e.g., brainstorming with butcher's paper, voting) and individual, anonymous activities (e.g., priority‐setting, rating the feasibility of solutions to target food insecurity). This mixture of group and individual activities was designed to foster collective participation while ensuring all voices could be heard. Co‐facilitators also rotated among tables during small group discussion to guide this process. However, it is possible that small group discussions may have been biased towards HCP or PWLE perspectives in some instances. Alternative options for participating were also provided (e.g., writing in individual notebooks instead of shared butcher's paper).

Implementation switched from a workshop format to focus groups based on feedback from participants desiring small group discussion when discussing more technical concepts and offering the potential for ‘closed’ groups (e.g., only PWLE or HCP participants). Focus groups comprised between 1 and 4 participants per group and similarly involved guided discussion, as well as collective activities (e.g., drawing on and annotating a print‐out synthesised model of care to target food insecurity). Only one focus group was ‘closed’ (available only to PWLE), another five involved both PWLE and HCPs. The singular focus group with the four co‐facilitators (C. O. D., O. A.‐Y., S. T., and A. M.) focussed exclusively on reflecting on the experience of co‐design, rather than also evaluating the pilot intervention design, as in the others.

Capacity‐building was consistently woven throughout both workshops and focus groups. Each new concept (e.g., a ‘model of care’) was introduced by co‐facilitators and applied in a tangible example (e.g., a local youth mental health service) to scaffold understanding. This was then explored as a group, co‐defining it (e.g., ‘The way a health service is delivered’) and evaluating what might be missing from the way the concept was being considered (e.g., ‘How does a health service promote self‐efficacy?’). Use of jargon and acronyms was intentionally minimised to enhance inclusion and understanding.

#### 2.2.4 Context

During workshops, participant groups were intentionally mixed to enrich the diversity of perspectives during discussions and balance power through shared decision‐making. For focus groups, participants were offered the opportunity to participate in mixed or closed (e.g., HCP‐only) groups. Food and refreshments were provided that catered to dietary and cultural needs (e.g., vegan, halal), and flexible reimbursement through an electronic bank transfer was provided for time and transport (e.g., parking costs).

Importantly for contextualisation, O. A.‐Y. and S. T. were embedded in the health service; based at the centre 1 day a week for 6 months and involved in incidental activities (e.g., unloading food rescue van deliveries). This enabled a sense of connection with both PWLE and HCPs, as well as experiential knowledge to guide probing of specific practices within the mental health service. However, this also led to a potential for bias through imbuing explanations of cultural dynamics with their own experiences, discussing this among the research team throughout.

In addition, after completion of the co‐design process, in‐services on food insecurity were delivered to mental health clinicians by HCP participants without involvement from the research team. A staff working group was also formed at the mental health centre to address the issue, and it was added as a permanent agenda item to centre staff meetings.

#### 2.2.5 Co‐Facilitation

In addition to being guided by McKercher's framework [26], the practical suggestions of Harrison et al. [38] specifically for co‐facilitation were considered (e.g., individual role definitions) and practised (e.g., openness and debriefing) among the team to ensure equitable contribution.

Workshops were held at the University of New South Wales, Sydney, and were not recorded, due to concerns about this potentially limiting participation early in the co‐design process. Co‐facilitators met participants at a familiar location outside a nearby mental health centre and walked together to the university for those who felt more comfortable with this. Workshops were co‐facilitated by a psychologist member of the research team who had not been involved in the original research design or funding acquisition but had extensive experience in co‐design facilitation (A. M.) and a LE facilitator. The LE facilitator was a peer support worker in Workshop 1 and a member of the research team (C. O. D.) in Workshop 2. This co‐facilitation approach was chosen to centre a LE perspective and incorporate an external perspective, supporting reflexivity. In addition, two research team members (O. A.‐Y. and S. T.) scribed throughout, taking detailed paper notes to amplify LE and HCP voices, as practised elsewhere in co‐design research [36]. These notes included verbatim quotations without noting identifying information (e.g., participant group), with contextual information, for example:

[Speaking about what leads to food insecurity]: ‘A change in a social dynamic, a change in income, if someone can't work or needs an operation’

Focus groups were held at the adult community mental health centre or online. These were co‐facilitated by O. A.‐Y. and S. T., and all were recorded using Microsoft Teams, following discussions of comfort and consent about recording with participants. The change in co‐facilitation was partly practical (time constraints of the previous co‐facilitators), as well as to mitigate desirability bias when discussing co‐facilitation of workshops. PWLE were also offered the opportunity to participate in a focus group facilitated by a LE researcher if they felt more comfortable with this, though none chose this option. For the co‐facilitator focus group, this was principally facilitated by C. O. D., incorporating their own responses and reflections among the group.

Across workshops and focus groups (excluding the co‐facilitator focus group), LE co‐facilitators and O. A.‐Y. offered the opportunity for psychological support, which multiple participants accessed. This collective approach allowed co‐design activities to continue while individuals stepped out for support. Co‐facilitators and scribes also intentionally shared the same physical space as participants (e.g., sitting at the same tables rather than standing to deliver inputs) to help create a sense of equality [35].

### 2.3 Co‐Design Process Evaluation

#### 2.3.1 Research Team and Positionality

Two members of the research team identified as LE researchers, all identified as Australian, and half identified as women and half as men. Individual roles and positionality are described in Box 2 below.

Box 2. **Research team roles and positionality.**

**Table:** 

Name	Initials	Role(s)	Positionality
Oliver Ardill‐Young	O. A.‐Y.	Conceptualisation, Methodological design, Scribe (Workshops 1 and 2), Psychological support (Workshops 1 and 2; focus groups), Co‐facilitator (focus groups; structured reflection), Analysis, Writing – original draft	Oliver is a clinical researcher with a background in psychology and linguistics.
Catherine O'Donnell	C. O. D.	Funding acquisition, Conceptualisation, Methodological design, Psychological support (Workshop 2; focus groups), Co‐facilitator (Workshop 2; co‐facilitator focus group), Analysis, Writing – original draft	Catherine is a Lived Experience co‐design and consumer engagement specialist. They have qualifications and extensive experience in adult learning and workshop design.
Scott Teasdale	S. T.	Funding acquisition, Conceptualisation, Methodological design, Scribe (Workshops 1 and 2), Co‐facilitator (focus groups), Analysis, Writing – review and editing	Scott is a researcher with a background as a clinical mental health dietitian.
Alyssa Milton	A. M.	Conceptualisation, Methodological design, Co‐facilitator (Workshops 1 and 2), Analysis, Writing – review and editing	Alyssa is a psychologist and academic with extensive co‐design, qualitative, and mixed methods expertise and draws on both industry and academic experience in the mental health sector.
Aaron Schokman	A. S.	Analysis, Writing – review and editing	Aaron is a post‐doctoral researcher specialising in narcolepsy and Lived Experience of chronic and rare disease.
Jacqueline Huber	J. H.	Analysis, Writing – review and editing	Jacqueline is a psychiatrist and researcher with experience in older adult, emergency, and inpatient psychiatric suicide crisis care.
Philip B. Ward	P. B. W.	Conceptualisation, Supervision, Writing – review and editing	Philip is a researcher with experience in mental health and lifestyle interventions.
Jackie Curtis	J. C.	Conceptualisation, Supervision, Writing – review and editing	Jackie is a psychiatrist and researcher with experience in youth mental health.

#### 2.3.2 Research Procedure

In place of a ‘blanket’ ethics application, amendments were made for Workshop 2 and focus groups, to iteratively incorporate feedback on the experience of co‐design (e.g., switching to a focus group modality) [39]. Therefore, while agendas were constructed by the research team, they were adapted to what emerged from co‐design. Written participant summaries were also circulated to participants after each co‐design session, including information on how the session was experienced by all groups. These were discussed with each participant to provide a space for further questions and strengthen relationships.

The research team met weekly throughout the project to develop a strong shared understanding and bond. During these meetings, C. O. D. was supported in learning how to conduct qualitative analysis, and responsibilities were consistently reviewed and negotiated.

A Steering Committee was also formed after Workshop 2, comprising the research team and co‐design participants (a dietitian, a peer support worker and a participant from the PWLE group—recruited through a targeted expression of interest). This was formed as we realised we needed more leadership and governance from HCP and PWLE stakeholders, to ensure that the project remained grounded in the community's real needs and prevent any single perspective from dominating the discourse [40]. The Steering Committee reviewed the ethics application and materials for focus groups and provided guidance on how to disseminate and implement findings from the project. This led to new dissemination pathways through the mental health service and Consumer Advisory Groups, as well as refinement of focus group guides.

A structured reflection on power dynamics using Goodwill et al.'s [41] framework was also undertaken during two meetings (total 1.5 h) by two co‐lead researchers involved throughout the entirety of the research process (C. O. D. and S. T.), facilitated by O. A.‐Y. This was also recorded using Microsoft Teams.

#### 2.3.3 Data Generation

A purpose‐built survey was distributed at the end of workshops, which explored participants' experiences of the co‐design process and collected demographic details. Survey items were a mix of open‐response questions or rating the level of agreement with statements on Likert‐style scales. Design and interpretation of the demographic questions were informed by the Australian Standards [42]. In addition, the experience of co‐design was also explored in the final half‐hour of focus groups. Questions regarding the experience of co‐design in both the survey and focus groups were partially adapted from McKercher [26], as well as Pallesen et al. [43] and a resource by Metro North Health [44].

Field notes and scribed notes taken by co‐facilitators, all physical materials used as part of the co‐design (e.g., butcher's paper), recordings of focus groups and the structured reflection, and open‐response survey answers were collected as artefacts for qualitative analysis. Field notes, survey responses, and physical materials were transcribed in Microsoft Word and automatic transcriptions (focus groups, structured reflection) generated by Microsoft Teams were reviewed for accuracy against the audio recording, using conventional methods [45]. These were then collated into a combined structure by section of the co‐design session (e.g., ‘Workshop 1; blank slate solutions’). Typographical errors were not corrected, though identifying information (e.g., participants' names) was removed or generalised. Validity of scribed notes was established through crystallisation with the other artefacts and cross‐referencing with the co‐facilitators [46]. In addition, while not formally analysed, personal memos and notes from meetings among the research team were recorded and used to stimulate reflexivity.

#### 2.3.4 Analysis

Descriptive statistics were calculated for demographic data. Mean values were calculated for Likert‐style survey items using SPSS Statistics (IBM Corp., Version 27, Armonk, NY), with higher means indicating greater agreement.

We used reflexive thematic analysis for qualitative artefacts as it requires situated, reflexive, and collaborative researcher engagement in the analysis, methodologically consistent with the co‐design process [31]. We adopted a constructionist epistemology which conceptualises meaning as co‐constructed between participants and researchers [47, 48]. Analysis followed the six phases outlined by Braun and Clarke, with emphasis on reflexive dialogue and deep reflection within the research team. The steps included: (1) *Become familiar with the data*: As the research team were involved in the co‐design implementation, familiarisation occurred through this process and the ensuing transcription, checking this for accuracy and engaging in fortnightly meetings about themes; (2) *Generate initial codes*: inductive semantic and latent line‐by‐line coding in Microsoft Word of two de‐identified focus groups and the experience surveys were conducted independently by C. O. D. and O. A.‐Y. to generate early analytic reflection and develop a preliminary thematic framework. Three researchers involved at this stage allowed multiple interpretive perspectives to inform the developing analysis. Interrogation of semantic compared to latent coding was used as a tool to guide reflexivity in discussion about meaning imposed and constructed by each researcher; (3) *Search for themes*: Open coding of all artefacts was then conducted progressively by C. O. D. and O. A.‐Y., separately, using NVivo 14 Software; (4 and 5) *Review and define themes*: The framework themes were developed iteratively through two collaborative discussions with S. T. and A. M., with attention to areas of convergence and divergence in interpretation. J. H. and A. S. then completed independent coding on the same data set using the framework in NVivo 14 Software. Differences in interpretation at this stage informed narrative discussions, with coding comparison used as a prompt for reflexive dialogue [11]. This approach ensured that there were representative backgrounds in the analytic team, drawing on LE, clinician, and supporter backgrounds, which follows other co‐production [49] and qualitative [50] research methods. This collaborative approach to analysis supported reflexivity as it encouraged comparisons and sharing of diverse perspectives; (6) *Write‐up*: the results were written up jointly by C. O. D. and O. A.‐Y. and reviewed by all team members. A lay summary of findings was provided to participants. However, there was no formal opportunity for participants to give feedback other than through conversations with the researchers directly during this process of communicating back to the community.

Following the structured reflection, team meetings were used to collaboratively consider the data [34]. The transcripts were similarly, inductively, thematically analysed through semantic and latent line‐by‐line coding in Microsoft Word by O. A.‐Y. with review by A. M.—as a researcher independent to the reflection to support reflexivity [48]. The matrix table with supporting data was collaboratively generated by C. O. D. and O. A.‐Y., and all co‐authors reviewed the final version.

## 3 Results

### 3.1 Survey Data

Survey data were available for *n* = 7 PWLE and *n* = 7 HCPs for Workshop 1 and *n* = 6 PWLE and *n* = 5 HCPs for Workshop 2. Items regarding participant understanding of and desire to participate in co‐design are reported in‐text below, while items related to the experience of the process are displayed in Figure 3.

**Figure 3:**
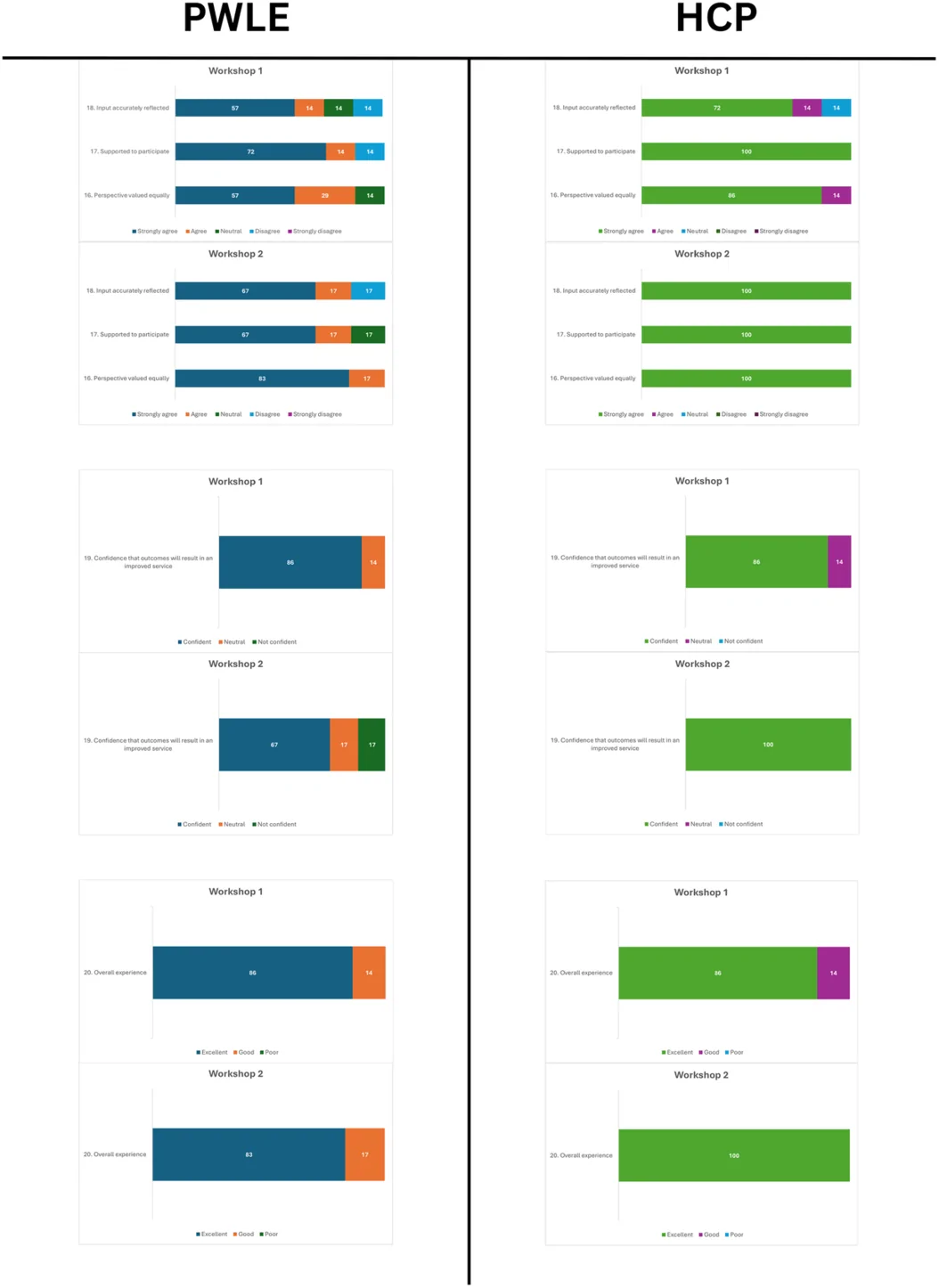
Survey results related to the experience of co‐design.

All (100%; *n* = 13 of 13) PWLE felt that their understanding of co‐design improved after each workshop, while 71% (*n* = 5 of 7) of HCPs felt their understanding improved after Workshop 1, and 100% (*n* = 6 of 6) felt it improved after Workshop 2. Eighty‐six percent of both PWLE and HCPs (*n* = 6 of 7, both groups) said they received sufficient information to understand Workshop 1's purpose, and all PWLE and HCPs (100%; *n* = 6 of 6 and *n* = 5 of 5, respectively) said they received sufficient information to understand Workshop 2's purpose. All PWLE stated that they were ‘very likely’ to participate in future co‐design after each workshop (100%; *n* = 7 of 7 and *n* = 6 of 6). Eighty‐six percent of HCPs stated that they were ‘very likely’ (*n* = 6 of 7), and 14% (*n* = 1 of 7) were ‘somewhat likely’ to participate in future co‐design after Workshop 1, and 100% (*n* = 5 of 5) stated that they were ‘very likely’ to participate in future co‐design after Workshop 2.

### 3.2 Qualitative Themes

The analysis generated three broad analytic categories: (a) ‘doing’ co‐design; (b) experience of co‐design; and (c) impacts of co‐design. Within these were clustered 12 themes, presented in Figure 4 below.

**Figure 4:**
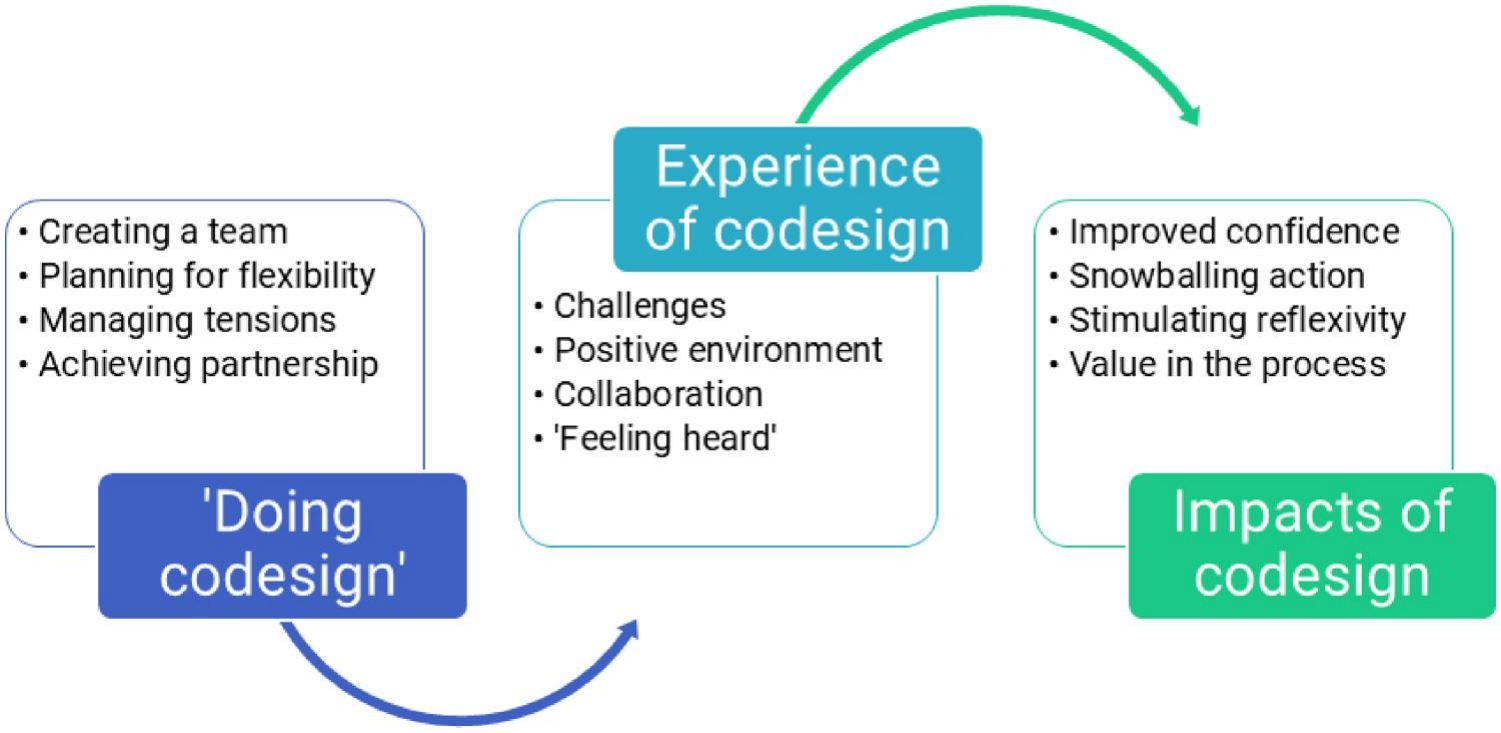
Qualitative themes.

#### 3.2.1 ‘Doing Co‐Design’

This category related to creating the conditions for co‐design and the active manifestation of participation within co‐design sessions. Captured within this category were themes of creating a team, planning for flexibility, managing tensions—in terms of power dynamics and expectations—and achieving partnership through building capability and feedback loops.

##### 3.2.1.1 Creating a Team

Which voices were important to be involved in the co‐design process was a core theme. Incorporating a diversity of perspectives for the project, both as participants and co‐facilitators, was highly valued for creating a team. In particular, LE co‐facilitation was viewed as creating space for PWLE to engage:

It's less confronting if there's someone else with lived experience helping to lead.

– PWLE

Collaboration between different groups was also felt to enhance the purposefulness of the co‐design:

I liked the collaboration and I actually like that there's the different backgrounds and skills giving input because then you [sic] less likely to have gaps and you're more likely to be able to design something that's more effective for a majority rather than a minority.

– PWLE

Within the research team, a diversity of backgrounds was also highlighted as key to creating a feeling of partnership. Continual check‐ins about the degree of power held by different members throughout the research cycle, taking time for joint reflection, and teaching each other new skills were seen as instrumental processes to engender a sense of ‘being a team’:

We collaborate really well together. We had role definitions but still overlay the collaboration. As a team we created an environment where people felt comfortable and open to their opinions, being heard and listened to.

– LE Co‐facilitator

##### 3.2.1.2 Planning for Flexibility

In‐depth planning of co‐design sessions, because of the need to incorporate the variety of perspectives in an equitable way, was identified as crucial. Key practical considerations for PWLE were the accessibility, both physically and psychologically, of the location: ‘it was a big barrier for [PWLE] to come to the university but it was also a big moment of “I'm here,” so it wasn't necessarily a bad thing’; and timing of sessions: ‘there's a lot of people that take medications in the evening and then they're groggy in the morning’. Multiple run‐through sessions within the project team prior to workshops also enabled flexibility in the roles of the research team (e.g., the last‐minute change in co‐facilitation of Workshop 1) and improved communication with participants:

The different exercises we did, because we had thought a lot about them and really wanted a purpose with them, we were able to communicate […] the nature of them very, very clearly so that people could really engage with them.

– LE Co‐facilitator

##### 3.2.1.3 Managing Tensions

The open‐ended and new process, as well as the different perspectives and backgrounds of participants, were constructed through our analysis as points of tension in the co‐design. Two key sources of tension were experienced: power dynamics and expectations of co‐design.

###### 3.2.1.3.1 Power Dynamics

Power imbalances between the co‐facilitators, HCPs, and PWLE, with co‐facilitators and HCPs experiencing greater power than PWLE, were seen as something inescapable:

It's not that utopia [where there are no power imbalances].

– Peer Worker

However, several strategies to manage power dynamics and resolve these tensions were identified. These included building a sense of shared aims, co‐facilitators expressing vulnerability about a lack of understanding and recognition of different types of expertise, reducing the length of co‐design sessions, and working in smaller groups:

[Shorter sessions] just spaces out everyone–I dunno–getting their dominance over the group.

– PWLE

All of you […] had a very person‐centred approach, and I think [the topic expert] said: “Now, I don't know who made that comment, [but] I don't really understand it.” You know that that sort of stuff of just […] made it really relatable […] and gave people the confidence to speak.

– Peer Worker

[Power felt more equal because] I think we all have the same goal.

– PWLE

###### 3.2.1.3.2 Expectations

Participants' expectations around the requirements and length of commitment to the co‐design process, as well as the guaranteed benefits and outcomes, were viewed as tensions. Clear communication about what co‐design would involve (the kinds of tasks, potential distress and emotional content, the length of sessions, and what could be realistically achieved in what timeframe) was identified as highly important for managing this:

[Screening and recruitment] was really helpful because people came in knowing what to expect. Knowing what was going on and knowing that they were being held by this lovely human, you know the human aspect, you can't underrate it with the sensitivity of our clients.

– Peer Worker

[The workshop was] too long for everyone, too much information.

– PWLE

[I'm] hopeful [about the project] but red tape means uncertainty.

– HCP

##### 3.2.1.4 Achieving Partnership

Beyond who was in the room in creating a team, achieving partnership related to the processes by which a connection was formed between participants and the co‐facilitators. This was seen as being underpinned by processes which built the capability of and rapport with participants, and the creation of feedback loops.

###### 3.2.1.4.1 Building Capability and Rapport

Partnership between the co‐facilitators and the participants was seen as stemming from the interactions prior to sessions (such as screening, detailed above), which developed trust, as well as description of key concepts (e.g., co‐design) that were carried forward, and modelling an informal environment:

Even if the facilitators don't need a break, even if they go out to stretch their leg, take it in turns to go out to stretch that leg […] it helps the consumers feel less noticeable if they need to do the same.

– PWLE

[I] loved the co‐facilitator's explanation of co‐design.

– HCP

In addition, working in small groups and including the co‐design team as participants in activities were seen as building a supportive team dynamic:

Having the person as the topic expert just come in and be completely open as well, like without ego […] coming around and sitting at the table, I just genuinely don't think it felt for people like they were being watched or like they had to be more articulate […] they could just speak to him as someone else at the table.

– LE Co‐facilitator

Across the two participant groups, enthusiasm for collaborating on activities and engaging in respectful sharing of lived experiences were highlighted as key processes for building connection:

Exploring “how does food insecurity make you feel?” […] we all shared ideas and were respectful of each other's point of view.

– HCP

[When people encourage you to write down what you have said it] makes you feel high and gives you a dopamine hit, and you're so good.

– PWLE

###### 3.2.1.4.2 Feedback Loops

Communicating information (e.g., the method for synthesising brainstorming activities, the results from prioritising solutions) in clear, accessible ways to the participants, coupled with seeking feedback on this feedback itself (e.g., asking about learning styles), was identified as a key mechanism for engendering feelings of collaboration. Creating this kind of dialogic relationship was seen as a form of recognition that people required different ways of understanding, ensuring transparency about the process, and demonstrating empathy:

When you come back to us with what you've got, you can see that you've listened.

– PWLE

I think [that receiving results summaries, people felt] a mixture between relief and happiness, that because we were giving the feedback it didn't seem […] as though they'd opened themselves out and said all this and then nothing had happened.

– Co‐facilitator

Asking about learning style […] yeah, that'll help.

– HCP

I found it very easy to follow [the results summaries], even though I get distracted easily, with the colours […] very comprehensive.

– PWLE

#### 3.2.2 Experience of Co‐Design

This category relates to how participation in co‐design was experienced, in contrast to the active processes (i.e., the ‘doing’) through which it was conducted. This comprised themes capturing challenges, the experience of a positive environment, a sense of collaboration, and of ‘feeling heard’.

##### 3.2.2.1 Challenges

The majority of participants across both groups felt that the tension of power imbalances was a key challenge. Though, as described above, strategies such as rotating scribing during brainstorming and collective decision‐making were felt to be effective in reducing imbalances. Consequently, while there was an awareness of power as ever‐present, there was a general sense across groups of its accumulation by any one group being prevented and neutralised:

With the different power dynamics, there wasn't the power in the room to grab onto it.

– Peer Worker

However, two PWLE felt that their input was not acknowledged by other PWLE or that they were talked down to by HCPs—with HCPs assuming a status of superiority:

When you're […] doing the right thing and writing on the butcher's paper and … you talk with other [PWLE] and they're not acknowledging, you [feel] that it's not a good point.

– PWLE

I got a bit of upset because one worker said to me said “Oh well, I make sure I budget for my children” because […] I had to get food vouchers for my children, and I felt that [that was] so insulting […] it really had me thinking “I don't want to go back to that [co‐design] again.” […] My problems that I had were just personal […] you're gonna get that amongst the group […] I still felt like I could have input and everything like that.

– PWLE

Within the example above, the PWLE vividly articulated the sense of being looked down on by an HCP participant and their intense discomfort in response—highlighting the felt power imbalance. They communicated this to co‐facilitators within the workshop during a break and engaged with the psychological support offered, returning to participate in the workshop and focus groups. Co‐design as a way of working together and addressing power imbalances were also collectively re‐iterated by the co‐facilitators. The participant's final comments relate back to the inescapability of tensions in power dynamics, but the capacity to actively manage these and maintain inclusive participation.

Other challenges included the length and timing of workshops and the use of technical jargon:

[At] 3 o'clock I get that big crash with energy.

– PWLE

In general, yes, I think people were engaged. Sometimes [with] jargon or towards the end before breaks people did struggle a bit.

– PWLE

Workshops beginning early in the morning were experienced as a key issue for some PWLE participants, due to side effects of drowsiness from the medication they were taking. The long duration of workshops was also exhausting and cognitively draining for some. While jargon and technical language were found to be helpful when there was an in‐depth collective explanation or definition (e.g., ‘a model of care’ or ‘food insecurity’), the volume of new terms and those introduced with minimal explanation were underscored as a barrier to meaningful engagement.

##### 3.2.2.2 Positive Environment

The overall atmosphere of the co‐design was experienced as stimulating, relaxed, and accommodating. Contributing factors to this were construed as the mix of different backgrounds involved in the process, reimbursement for time and transportation, and sharing food and breaks together:

Making sure people are getting reimbursed is important’ – Peer Worker

All the different types of people being in the room from the client to the case manager, dietitian and the different sorts of people in the room [was interesting].

– PWLE

The food was good! Everyone liked the food.

– Peer Worker

[There was a] relaxed atmosphere, inclusive, flexible style and warm facilitators.

– HCP

##### 3.2.2.3 Collaboration

A salient aspect of the experience of co‐design perceived by HCPs was a feeling of collaboration with PWLE:

I thought the fact that we were all there together, really […] blurred all those boundaries so we were able to just sit and chat like human beings.

– HCP

It's so refreshing I think to have [PWLE] come in, be part of something and actually just express themselves.

– HCP

As highlighted in the quotes above, this feeling was seen as emerging from the unique context of working together on a shared goal, with perceived removal of traditional roles in the dynamic of HCPs and PWLE (i.e., with HCPs setting an agenda). HCPs articulated a sense of shared honesty and freedom of expression through co‐design, which is often lacking within these traditional roles.

##### 3.2.2.4 ‘Feeling Heard’

Consistent with this, PWLE expressed feeling understood, having their expertise recognised, and experiencing genuine participation in decision‐making about health care:

I'm sure I speak for a lot of consumers [in] actually feeling heard instead of just a label or a tick box […] and actually being able to be part of something for us rather than something done to us.

– PWLE

It was really validating and empowering to see the accomplishment of something to help other people that has the support of clinicians and consumers in the creation of it.

– PWLE

In particular, this was felt to be in stark contrast to previous participation in more consultative forms of service user engagement. This sense of being heard was closely tied to an emergent empowerment, confidence and self‐efficacy.

#### 3.2.3 Impacts of Co‐Design

This category encompassed themes and sub‐themes relating to personal and emotional outcomes of being involved in co‐design (‘improved confidence’ and ‘self‐awareness’), changes in the healthcare system (‘snowballing action’ and ‘clinical practice’), community, and value in the process of co‐design itself.

##### 3.2.3.1 Improved Confidence

PWLE felt increased confidence to influence change and improved self‐esteem following participation in co‐design:

It gives me the feel [sic] at the moment that I'm not the most trivial man in the world.

– PWLE

It's just having the ability to input, just as a person who doesn't do much and isn't, you know, doesn't work or anything like that. You know the ability to […] find my intelligence and put it in words, in something that I can talk about. You know, it makes me feel like, “oh, you know, my mind's still ticking alright”.

– PWLE

This new confidence was traced to the experiences of being listened to and valued for their input, often challenging internalised negativity about the worth of their contributions. It was also described as progressively growing throughout the ongoing participation in the co‐design process.

##### 3.2.3.2 Snowballing Action

The developments and actions taken by HCP participants, independent of the research team (e.g., delivering in‐services, forming a working group), were viewed as an example of the expanding impact of the co‐design process. That is, the co‐design acted as a catalyst for ongoing action within the service, contributing to increased awareness of food insecurity and supporting changes in practice beyond the original scope of the project:

The momentum of just to start talking about food security led to – in the absence of a research team – the clinicians and peer workers to develop their own working group and start testing their own ideas … [that] means that there's been a change.

– LE Co‐facilitator

##### 3.2.3.3 Stimulating Reflexivity

###### 3.2.3.3.1 Clinical Practice

Clinicians expressed feeling exposed to new ideas and ways of thinking, and peer workers perceived changes in how clinicians engaged with PWLE following the co‐design. We also saw an increased appreciation of the approaches of different mental health disciplines:

I get to look out of my frame and appreciate, you know, disciplines and the restrictions a lot of disciplines have.

– Peer Worker

[Co‐design] just brings so much … enrichment and humanness and things I probably wouldn't even consider […] it's like “Wow, didn't think of that”.

– HCP

It changes clinicians. Sometimes it is just their language is changed. Their perception of who they are is changed, but yeah, I see a really big change. They don't necessarily become a different clinician, but they definitely have different eyes.

– Peer Worker

###### 3.2.3.3.2 Self‐Awareness

PWLE emphatically spoke about increased understanding about their relationship with food insecurity and mental health, especially through sharing experiences with other PWLE, HCPs, and researchers:

It makes you think about […] stuff like, yeah, you haven't pinpointed it in yourself yet.

– PWLE

I never thought about [food insecurity] before we did these groups.

– PWLE

It makes you aware of things that you're aware of, but not conscious of […] [hearing] other people's opinions, like “I feel like that too”.

– PWLE

We conceived of this as an insight which developed progressively throughout the iterative process, through the guided practices within sessions, conversations in friendships between participants which came about through meeting as part of the co‐design, and their own private reflections.

##### 3.2.3.4 Value in the Process

Co‐design was seen as a positive force in and of itself, as a beneficial process to be part of and through the empowerment of PWLE:

You know when [co‐design] works, it just works beautifully, you know, and [a PWLE participant] is still apologising for […] having to go home [early] because he was falling asleep at 3 o'clock in the morning so excited to try get [to the workshop]! (laughing) […] That's just a lovely outcome in itself.

– Peer Worker

And even having the workshops and having this time […] what you are doing is really good.

– PWLE

The [PWLE] that I know that were there that I'm now engaged with, they still carry the essence of empowerment that they were consulted, OK and that holds power. They hold that as a memory and it is very powerful.

– Peer Worker

As the examples above encapsulate, we understood our participants as identifying that there was intrinsic value in the process of co‐design through a profound sense of empowerment, which was long‐lasting, instilling energy and dignity. In particular, this theme underscores the harms of focussing on impact alone [18]. In addition, the process was seen as healing relationships and building trust between the mental healthcare system and PWLE:

Given the number of people who've had trauma either predating mental health challenges or triggering and contributing to them or caused by the way the mental health service has operated, more so in the past than recently, it probably would have actually helped [PWLE] to have more confidence in some of the services.

– PWLE

### 3.3 Power Reflection

Table 2 details the critical reflection of co‐researchers on different domains of power throughout the co‐design process.

**Table 2:** Critical reflection on domains of power dynamics.

Domain	Reflection
*Privilege:* ‘The type of power that allows a stakeholder to influence a design process due to an unearned advantage based on their social position or identity’.	There was a lack of cultural diversity among the research team, and all spoke English as a first language. While multilingual healthcare professionals and Aboriginal Health Navigators were involved in recruitment, and culture was considered in planning (e.g., offering options for interpreting, catering), cultural safety was not foregrounded in the design process, and this may have led to unintentional exclusion: ‘[It's] the same groups that get left behind with all the stuff that we do, because we don't think about how accessible what we're doing is’.
	A LE researcher co‐leading this project was felt to be a positive privilege, in the power to connect with and understand the experiences of participants with lived/living experience: ‘They're my people and I'm one of theirs’.
	The academic backgrounds of research team members and the university setting of workshops were a salient facet of privilege: ‘People looked to me as an authority … I think it affected people in thinking that they had to achieve something for me, rather than co‐designing something better for everyone’.
*Access:* ‘The ability to influence who is included and who is excluded from a service design project’.	From the start, a LE researcher co‐led all parts of the research process to ensure this perspective throughout the project, consistently reflecting on: ‘What [are] the important things we need to consider when we're looking at designing workshops and bringing in people with lived experience? … How can we make the experience for those individuals collaborative and safe?’.
	As relationships formed through the project developed, decision‐making about research design (e.g., creating question guides, adapting to focus groups instead of workshops) was also diffused through the formation of the Steering Committee: ‘We started thinking a bit more broadly and we needed different voices’.
	Eligibility criteria of a diagnosis of a severe mental illness to participate in the co‐design was based on previous prevalence research demonstrating food insecurity as an issue for this group. However, potential exclusion emerged through the focus groups, and criteria for the pilot intervention implementation were modified accordingly, recognising that: ‘Anyone experiencing mental illness is likely at higher odds of experiencing food insecurity as well’.
*Goal:* ‘The ability of designers to initiate and frame the design project, including the way in which problems are defined, goals are chosen, and the design project as a whole is structured’.	The project was co‐led, with limited constraint from grant funding structures over defining the processes, timing or highly specified outcome(s): ‘[The project was] open to change, probably just not the desired overarching concept of the outcome that we're after, which was to sort of explore food insecurity and generate solutions from co‐design’.
	The problem of food insecurity was also collectively defined through the early stages of co‐design: ‘Lots of discussion, lots of listening, lots of learning from each other’.
*Role:* ‘The ability to influence the roles that different actors—those who have already been given access to the project—will assume during a design project’.	Roles among the project team changed throughout the project, from co‐facilitator, researcher (e.g., in analysis), or content specialist (e.g., food insecurity), with these roles shaped by capacity at different stages and expertise, though decision‐making was always shared: ‘We all took part in all of it, but we had different degrees of how involved we were’.
	Decision‐making was frequently transferred from the project team to participants (e.g., through individual rating of potential solutions) and, where decisions were made by the project team (e.g., synthesising ideas between workshops), these were reviewed by participants: ‘We brought it all back together again in the focus groups to really check in on that interpretation and prioritising what the actual findings were’.
	Outside of those who joined the Steering Committee, participants were not involved in design or analysis, due to a lack of resources, confidence, and support structures: ‘I've never done it before so there's a little bit of fear factor in there … how do you facilitate that? … the way we're doing it is already fairly resource and time‐intensive’.
*Rule:* ‘The ability to establish the way that actors included in the design network will work together’.	Addressing power and role dynamics at the start and throughout of every co‐design session, and reassuring participants about ‘sitting in the grey’, likely amplified the voices of lived experience participants: ‘It raises in people's minds to stop … and think about, you know, talking over others, letting people finish what they want to say, respecting their lived experience and their story and their narrative’.
	Norms from traditional power roles still prevailed, though: ‘When you let the tables [in workshops] do their own thing, naturally a clinician would take the paper and the pen, and it automatically feels like a transition of power’.
	However, establishing collective decision‐making processes (e.g., voting), covering different options for communicating (e.g., individual notebooks or scribing), and having the project team facilitate on the same physical level (e.g., sitting at the same table) all were seen as rebalancing this power: ‘it dissipates it … it just kind of goes’.

## 4 Discussion

By examining how co‐design strategies operate in practice and explicitly interrogating power dynamics, this paper advances understanding of the mechanisms through which co‐design shapes collaborative processes and generates impacts across individual and system levels. Several key elements of ‘doing co‐design’, in planning for a flexible process, creating a team, and achieving partnerships, were perceived as contributing to the impacts of overall positive experiences and personal growth. The main challenges which were generated through our analysis related to managing expectations of the inherently uncertain process and power imbalances. Co‐design was also seen as having translational effects on clinical culture and catalysing reform within the health service.

With the growth of interest in participatory methods [11], evidence supporting key processes of co‐design has grown. We found that managing tensions around people's expectations of co‐design was central to creating conditions for collaboration between different participant groups, ensuring that each person felt like they had the skills necessary to contribute meaningfully to what could be an unfamiliar and evolving process. This echoes two recent studies exploring the experience of co‐design, one of which involved researchers and HCPs [43] and another involving HCPs, young people at risk of suicide and their carers [51]. Key to this is generating a shared understanding of what co‐design is, and transparency about the challenges that are likely to occur, such as uncertain timelines and outcomes.

Our findings also reflect established positive impacts, such as those captured in the review by Sheikhan et al. [16], including PWLE feeling heard and experiencing increased confidence, a sense of reciprocal learning in collaboration between PWLE, HCPs, and researchers, and enhancing the diversity of perspectives in the research environment. Where studies in the review noted that the participatory approach of engaging with LE informed a range of ideas and outcomes within the project itself, we found that our co‐design process had a snowballing effect on the health service more broadly. This indicates that co‐design may extend beyond project‐level outcomes to influence clinical culture and organisational priorities.

Furthermore, reflexivity is considered principally as an impact on research quality in Sheikhan et al.'s [16] review, while we found that the co‐design process engendered self‐examination of HCP's clinical practice. This included reflecting on the restrictions of a medical model compared to peer support and adopting more recovery‐oriented language. Given the well‐documented challenges of effecting culture change and reform within mental health systems, with a lack of ownership over innovation a key impeding factor, this highlights the broader influence of co‐design as a methodology extending beyond an individual project. Similarly, PWLE described increased self‐understanding and a sense of transforming their narrative, which emerged in several studies included in Sheikhan et al.'s [16] review.

These findings suggest that, where the impact of co‐design is measured, measurement should move beyond the three R's to encompass a broader range of impacts, as articulated in Peters et al.'s [52] recent Co‐Design Evaluation Framework. Although this framework was published after the conclusion of our project and was therefore not used during our analysis, our findings align closely with the framework's tree metaphor [52]. ‘Below‑ground’ components relating to people, groups, and research processes in context are reflected in themes relating to ‘doing co‐design’, and the experience of participation in our paper's co‐design appears to underpin ‘above‑ground’ outcomes at the people and system levels (e.g., snowballing action in our paper), including sustained organisational attention to food insecurity.

Moving beyond impacts to consider the ethical imperative of participation [18], the value of involving PWLE as both an empowering force and in forming positive relationships between PWLE and the mental health system was evident in our paper. Although our research was perceived by all participants as having been improved through the co‐design process [25], one PWLE strongly voiced feeling upset by an HCP during a workshop, questioning whether they wanted to continue participating. This occurred, as in Kehoe et al.'s study [51], despite multiple strategies employed to address power imbalances during the co‐design workshops. The participant engaged with the psychological support offered as part of the co‐design. They returned to participate in focus groups and ultimately expressed experiencing personal growth and multiple other benefits of taking part. Their experience reinforces the critical need to create safety in the co‐design environment to be able to communicate those feelings and provision of support options [53]. Aside from such tangible instances within the co‐design itself, our framework reflection illustrates ‘unseen’ power imbalances which may further exacerbate inequity, particularly in terms of potential exclusion of First Nations or culturally and/or linguistically diverse peoples [22].

### 4.1 Practical Recommendations

A key recommendation is to engage in power reflection at the outset and throughout the co‐design process, resonating with another recent Australian study evaluating co‐design within an inpatient setting [53]. This will assist in ensuring ongoing awareness throughout [51] and mitigate risks of exclusion and stigmatisation. In addition, offering multiple mechanisms of contributing (e.g., through scribing collectively, individually or participating in discussion) and anonymous methods will also help to mitigate these risks [35]. We aligned our process with valuable existing recommendations for co‐design [38, 43], including with PWLE of severe mental illness [54]. Specifically for engaging with PWLE of severe mental illness, we would add the need to explicitly consider the psychological safety of the location [13] and to plan sessions for times of the day which limit the impact of potential side‐effects of medications.

Moving forward, significant work remains to be done in the co‐design space, particularly in deepening our understanding of underlying mechanisms to strengthen the theoretical foundation and advance consistent best practices. Future research should focus on applying structured guidance for evaluating co‐design in health research and consider the ethical imperative for these evaluations to also be co‐produced [18, 22, 55]. This approach will improve measurement quality, provide clearer insights into co‐design's impact, and enable meaningful comparisons across studies [52].

### 4.2 Strengths and Limitations

Limitations of our paper include a lack of iterative structured power reflections, which may have ameliorated the potential exclusion of First Nations or culturally and/or linguistically diverse peoples and aided greater identification of power imbalances within co‐design sessions. In addition, while we explored experiences anonymously through surveys and directly probed power during focus groups, utilising Goodwill's framework [41] in these methods would have enhanced understanding of these dynamics. Future studies should incorporate these components. Not all participants who took part in co‐design participated in focus groups, and we did not specifically probe experiences as part of the Steering Committee, also limiting a comprehensive understanding. In addition, a potential limitation is the lack of consistency in participation across co‐design sessions and the relatively small number of participants for some of the focus groups (e.g., *n* = 2 participants) [56].

Our paper has numerous strengths. While Goodwill's framework [41] was not used to its full potential, we explicitly interrogated the power dynamics of co‐design [18] and explored negative experiences and their perceived causes. We also described transparently [15] the levels of participation of the co‐design team and of PWLE, as well as processes involved in co‐design, making explicit strategies which relate to core principles [26]. Several of these strategies likely mitigated challenges experienced in other co‐design studies, such as the use of rotated scribing and capturing direct quotes during workshops to minimise ‘filtering’ of PWLE's contributions by scribing HCPs [51]. LE expertise was involved in all stages of the research project, engaging in mutual learning and supporting academic service user/survivor researcher development. Our approach to co‐design and co‐facilitation was also aligned with and built on best‐practice guidelines [38, 43, 54], which future studies can also use.

## 5 Conclusions

Our paper contributes to the understanding of how co‐design is enacted and how it is experienced by PWLE, HCPs, and researchers. Through exploring power dynamics as an ever‐present but navigable force within participatory research, we provide insights into how to enhance collaboration between these participant groups. The current findings complement the emergent literature on the impacts of co‐design and suggest new, diffuse effects. At the core, our paper demonstrates the intrinsic value of the inclusion of PWLE in research.

## Author Contributions

**Oliver Ardill‐Young:** conceptualisation, formal analysis, investigation, methodology, project administration, resources, validation, visualisation, writing – original draft, writing – review and editing. **Catherine O'Donnell:** conceptualisation, formal analysis, investigation, methodology, resources, writing – review and editing. **Alyssa Milton:** conceptualisation, formal analysis, investigation, methodology, resources, writing – review and editing. **Jacqueline Huber:** formal analysis, writing – review and editing. **Aaron Schokman:** formal analysis, writing – review and editing. **Philip Ward:** conceptualisation, supervision, writing – review and editing. **Jackie Curtis:** conceptualisation, supervision, writing – review and editing. **Scott Teasdale:** conceptualisation, formal analysis, investigation, methodology, project administration, resources, supervision, validation, visualisation, writing – original draft, writing – review and editing.

## Ethics Statement

This study was approved by the South Eastern Sydney Local Health District Human Research Ethics Executive Committee (2022/ETH02327).

## Consent

Data were obtained directly from participants who completed a written informed consent procedure.

## Conflicts of Interest

The authors declare no conflicts of interest.

## Supporting information


Supporting File 1

Supporting File 2

Supporting File 3

Supporting File 4

Supporting File 5

Supporting File 6

Supporting File 7

Supporting File 8

Supporting File 9

Supporting File 10

## Data Availability

Data sharing is available upon reasonable request and approval from the South Eastern Sydney Local Health District Human Research Ethics Executive Committee.
