# Supplementary material for: ‘That Essence of Empowerment’: An Exploration of People Living With Mental Illness', Healthcare Professionals', and Researchers' Experiences of Co‐Design
Source: Health Expect. 2026 Oct 15;29(5):e70888. doi: 10.1111/hex.70888 (PMC13670414; doi:10.1111/hex.70888)
Supplement: Supplementary file 1 [file HEX-29-e70888-s003.pdf]

What level of participation can your project achieve?

Use this checklist to assess the participation options for your project.

Instructions: Work through one column at a time, and work from the top down. Identify the highest level you can reach in each column and tick that option. The lowest row with ticks is the level of participation that your project can achieve.

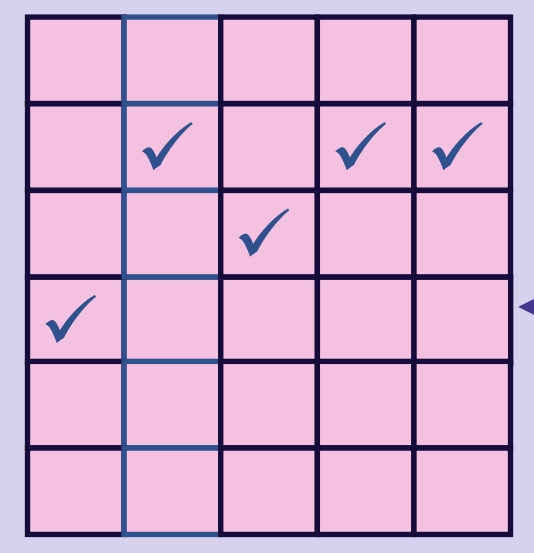

Participation level	How many consumers are involved & how?	Who decides the project scope, defines the problem & sets the outcomes?	Who will deliver the project?	Scope of engagement	Process	Decision making during the project	Budget	Timing
		Who decides the problem being addressed? Who decides on the project scope and outcomes?		*What is the limit of engagement?*	*How will processes redress power imbalances?*	*How will decisions be made during project work?*	*Do you have budget to pay for consumer participation?*	*How much time do you have to allow for consumer participation?*
**Consumer owned**	☐ All consumers + deep involvement	☐ Consumers will initiate the project and make all decisions. We provide the funding.	☐ Consumers	☐ Consumer enterprises are accountable for all aspects of project planning & design	☐ The process is outside of us and inside consumer enterprises.	☐ Consumers will make all the decisions (which may include inviting non-consumers to be part of the process).	☐ We have budget to fund these initiatives.	☐ Consumers told us the timelines they needed
**Consumer led**	☐ High majority of consumers + deep involvement	☐ Consumers lead decision making and have the final say.		☐ Consumers lead project planning, design.	☐ Consumers lead the process.	☐ Consumers will define how decisions are made during the project.	☐ Consumers manage the project budget.	☐ We have time to allow for consumers to lead the project. We understand the process may be different and take longer.
**Co-production**	☐ Majority of consumers (or at least 50%) + deep involvement	☐ We'll work with consumers to make these decisions together, with consumer views being prioritised.	☐ Consumers will be employed to be part of delivering the project	☐ Consumers share equally in project planning & design. Nothing starts until consumers have joined.	☐ Consumers form a majority of working group or committee memberships.<br>☐ Consumers have an equitable status and resources to others.<br>☐ Proactive steps are taken to redress power imbalances & seek equity	☐ Open & critical discussions are had about differing views.<br>☐ Decisions are not made until the majority of consumers agree	☐ We have budget to pay all involved consumers for their time and relevant expenses, and pay is commensurate with expertise.	☐ We have time to allow for in depth, challenging & innovative work with consumers.
**Co-design**		☐ These decisions are set & cannot change.	☐ Consumers may or may not be part of delivering the project	☐ Planning is already done.<br>☐ Consumers are partners in project design.				
**Engagement**	☐ Low number of consumers + deep involvement			☐ Consumers will be one of many inputs to design. Consumer views will be sought but necessarily incorporated.	☐ Consumers will be one of the groups represented in project work.	☐ Consumers will have a say in decisions.	☐ There is only enough budget to pay a small number of consumers for their participation.	☐ Timelines have some flexibility, but not a lot.
**Consultation**	☐ High number of consumers + shallow involvement				☐ We will ask consumers what they want and need, and how we should go about the project – but this will be outside of the project work itself.	☐ Consumers are not part of decision-making. But we will take what consumers want into account when we make decisions.	☐ There is no budget to pay consumers for participation.	☐ Timelines are tight and cannot be changed.
**Informing**	☐ High number of consumers + no involvement			☐ We will tell consumers what we've planned & designed.	☐ We will seek consumer feedback after the project.	☐ Consumers are not part of decision-making.		
**Educating**<br>**Coercing**<br>**Excluding**	It is not recommended to operate in these levels for any work that affects consumers.							

This checklist is a working draft, developed by Indigo Daya. You are welcome to use and distribute it. Contact: indigodaya@gmail.com
