# Supplementary material for: ‘That Essence of Empowerment’: An Exploration of People Living With Mental Illness', Healthcare Professionals', and Researchers' Experiences of Co‐Design
Source: Health Expect. 2026 Oct 15;29(5):e70888. doi: 10.1111/hex.70888 (PMC13670414; doi:10.1111/hex.70888)
Supplement: Supplementary file 2 [file HEX-29-e70888-s001.docx]

| Timing | Section | Facilitator | Activity | Tasks | Resources |
| --- | --- | --- | --- | --- | --- |
| 10:00 –  10:15  (15 mins) | Welcome | K | - Acknowledgement of country and lived experience. - Intro Facilitation Team - Everyone Roles, AM, CO, OA and KB - Housekeeping – Facilities, Fire Exit and Payment - Agenda and Breaks - Support available | Presentation | Slide 1: Welcome  Slide 2: Acknowledgements  Slide 3: Agenda and Housekeeping |
| 10:15 – 10:35  (20 mins) | Introductions | A | - Your name, something about you | Activity | End intro |
| 10.35 – 10.40  (5 mins) | Project introduction | S | **Why are we here?**   - Brief overview to this project | Presentation | Slide 4: Why are we here? |
| 10:40 – 11:00  (20 mins) | The foundations:  - What is co-design?  - Working together? | K | **What is co-design? (10 mins)**   - Introduce key ideas about co-design. - The co-design processes. - *How does that make you feel, what do you think about this change in practice* | Presentation | Slide 5: What is co-design?  Slide 6: Building image |
|  |  | K | **Working together (10 mins)**   - Power – maybe share reflections about power, or a quote (e.g. ‘Power is like oxygen, people only notice power when they don’t have it…’ Mary O’Hagan) - Different types of power - *What might be some strategies to ensure you feel comfortable and safe to share your thoughts and experience.* - Expectations & working together – what does these mean for this group? | Presentation | Slide 7: Power quotes  Slide 8: Working together |
| MORNING TEA BREAK 11:00 – 11:20 (20 mins) | | | | | Slide 9: Coffee Break |
| 11:20 – 11:30  (10 mins) | Meaning of Food Insecurity (FI) |  | **What is your definition of Food Insecurity?**  Groups allocated as per tables | Small group activity - Definition of FI from each group  Nominate person to present back | Slide 10: What FI and what does it mean to you – Definition  Butchers Paper/Markers |
| 11.30 – 11.50  (20 mins) | Definition and description of FI |  | **FI description and Video**   - FI definition and impacts in Australia. - Check in on how this definition compares to persons experience? | Presentation | Slide 11: FI definition + Video |
| 11:50 – 12.10  (20 mins) | Understanding FI from your perspective |  | **What leads to FI and how does it make you feel?**  Break into groups: consumers and clinicians.  Questions   1. **What do you think leads to FI?** 2. **How does FI make you or others think, feel and act?**   Reflection and Discussion | Small group activity.  A facilitator for each group.  Nominate person from each group to provide feedback | Slide 12: What leads to FI insecurity and how does that make you feel? **Questions 1 & 2**  Handout - Feeling Words  Butchers Paper/Markers  Small group work and nominate a person to speak to your findings |
| 12:10 – 12:30  (20 mins) |  |  | Questions   3. **What Impact does FI have on your physical health?**   Reflections and Themes | Group activity  Butcher paper- each question on the wall participants use post it notes for thoughts and ideas | Slide 13: What impacts on your physical health and what support is needed? **Question 3**  Butchers Paper/Markers/ Post it notes. |
| LUNCH BREAK 12:30 – 1:15 (45 mins) | | | | | Slide 14: Lunch Break |
| 1:15 – 2:15  (60 mins) | Solutions activity 1 |  | **World Café Activity**   - World café process to start to co-design solutions program.   - Table 1 – Potential Solution #1   - Table 2 – Potential Solution #2   - Table 3 – Potential Solution #3 - 5 mins explanation, 15 mins at each table - 10 mins for facilitators to feedback key points | Small group activity.  One facilitator for each table.  **Pre-design**  Butchers paper with option heading, pros/cons, barriers/facilitators | Slide 15: Solutions  Facilitator to record on Butchers Paper  3 key ideas for solutions to be explored – 1 at each table. |
| AFTERNOON TEA 2:15 – 2.35 (20 mins) | | | | | Slide 16: Coffee Break |
| 2:35 – 3:05  (30 mins) | Solutions activity 2 & debrief |  | **Extended co-design of solutions.**   - Blank canvas ideas – waving a magic wand what could be done as solutions? Participants to describe any and all of their own possible solutions.   Debrief on World Café activity | Group Discussion | Slide 17: Magic Wand  Facilitator to record on Butchers Paper |
| 3:05 – 3:35  (30 mins) | Identifying FI in services |  | **Should mental health or community services routinely ask about food insecurity?**   - Should food security be routinely asked about or screened for? - Who should do it? How should they do it? When should they do it? Where should they do it? | Group activity | Slide 18: Service to ask about FI?  Butchers Paper/Markers/ Post-it notes  Butchers paper 1 – ask, screen or neither?  Butchers paper 2 – who, how, when, where |
| 3:35 ­– 3:50  (15 mins) | Survey |  | **End of workshop surveys**  Your Experience Survey | Hand out survey to participants | Slide 19: Evaluation  Survey Handout |
| 3:50 – 4.00 (10 mins) | Close |  | **Around the room closing exercise**  One word to describe how you are feeling, or your experience of today’ | All to participate | Slide 20: Thankyou |
