# Supplementary material for: ‘That Essence of Empowerment’: An Exploration of People Living With Mental Illness', Healthcare Professionals', and Researchers' Experiences of Co‐Design
Source: Health Expect. 2026 Oct 15;29(5):e70888. doi: 10.1111/hex.70888 (PMC13670414; doi:10.1111/hex.70888)
Supplement: Supplementary file 3 [file HEX-29-e70888-s002.docx]

# Reflexive Thematic Analysis Reporting Guidelines (RTARG) Virginia Braun & Victoria Clarke

Supplemental materials to: Improving the methodological coherence and reflexive openness of the reporting of thematic analysis in *Palliative Medicine*: A review of 20 articles citing Braun and Clarke.

These guidelines are intended for use by authors, and by reviewers, to facilitate thoughtful, knowing, and most importantly conceptual and methodological coherence in (the reporting of) reflexive TA (RTA). They should be contextualised alongside our other writing (and that of other scholars), where clarification is needed. Our works include: a discussion of common problems, and a question based tool for evaluating TA research for publication;^1^ a discussion of reporting ”standards” for reflexive TA;^2^ a discussion of quality in RTA;^3^ a wider critique of the popular qualitative quality “checklist” COREQ;^4^ and the development of a fully qualitative set of reporting guidelines.^5^

The RTARG consists of information across three columns: 1) advice for different aspect of a research report/approach to reporting RTA (written as instructive); 2) guiding notes for further explanation; and 3) things to avoid – often practices that are normalised in some contexts for qualitative research reporting, but are conceptually incoherent with RTA. Some of the items are descriptive, specifying material that authors should consider including when reporting RTA. Some are evaluative, requiring authors to critically appraise their report for methodological coherence and transparency. Not all list items will be relevant, depending on the research design. We encourage authors to make use of supplementary materials to meaningfully address these reporting guidelines.

| **Advice for aspects of the research report/approach to reporting** | **Guiding notes and further explanation** | **Practices, concepts and terminology to avoid** | **Use of the guidance in the manuscript** |
| --- | --- | --- | --- |
| **The Introduction**  NB: We prefer *Introduction* over *Literature Review* as a section heading, to capture the broader purpose of this section. | | | |
| ***Background and rationale*** | | | |
| Provide a robust context and rationale for the proposed research in the *Introduction*. | Can discuss existing research, theory, and the wider context; the researcher is understood as *entering a conversation* with existing scholarship. | Critiquing the methodological limitations of existing research from a (post)positivist/ quantitative standpoint; orienting a literature review to finding a “gap” that the  research fills. | We provide a deep contextual grounding within the introduction, entering into a conversation with activist movements, historical disempowerment and current literature. |
| Clearly articulate a research question – one that is methodologically coherent. | Can discuss refining an initially broader research question to a more specific one for the  paper. | Formulating research questions as hypotheses or expectations about what might be “found”. | We articulate the specific aims of the paper and research questions. |

| **Advice for aspects of the research report/approach to reporting** | **Guiding notes and further explanation** | **Practices, concepts and terminology to avoid** | **Use of the guidance in the manuscript** |
| --- | --- | --- | --- |
| **The Introduction (Continued)**  NB: We prefer *Introduction* over *Literature Review* as a section heading, to capture the broader purpose of this section. | | | |
| ***“Owning your perspectives”^6^*** | | | |
| Include information on guiding theoretical assumptions and other (e.g., explanatory) theory informing the use of TA. | Guiding (e.g., paradigmatic, ontological and epistemological) and other theory should be coherent with RTA. | (Post)positivism and (simple) realism. | We outline constructionist approach within the methodology section explicitly and adopt this stance in critique of a positivist focus on impact and outcomes alone. |
| Report in a way that is consistent with stated theoretical assumptions throughout. | Theoretical coherence is evidenced through the use of language and concepts (e.g., around theme development, research subjectivity, data interpretation), the treatment of data, and use of quality practices consistent with RTA. | Inadvertently “mashing-up” of RTA and (post)positivism/ realism (e.g., assuming data interpretation can be accurate and reliable) – without a clear rationale. | We consistently use language addressing a development process and research subjectivity. |
| Evidence methodological coherence/integrity in both the research and the report.^7^ | Theoretical assumptions, research questions, methods/practices of data generation, RTA, and specific orientation to RTA, purpose of research etc. all “fit together”, conceptually. | Ontological and epistemological confusion (e.g., claiming constructionism but focusing on lived experience and treating language as a transparent window onto this). | We explicitly address the co-construction of meaning, and processes of interpretation and generation. |
| Show evidence of reflexive practice. | Can discuss researcher professional or personal positioning and experience in relation to the topic, and/or participant group, and/or their role in shaping the research; use of reflexive journaling. | Evoking researcher bias (positivist), or even researcher influence, in a way that evokes it as possible rather than inevitable. | We describe positionality of each of the researcher’s and reflections on how our experiences and positionality interacted with interpretation (e.g., through the embedded researchers). |
| Write in a methodologically coherent style. | A first-person writing style suits RTA, as it “writes in” the researcher and contributes to situated and reflexive reporting. | A third person writing style – writing the researcher out of the research. | We write in a collective first-person. |

| **Advice for aspects of the research report/approach to reporting** | **Guiding notes and further explanation** | **Practices, concepts and terminology to avoid** | **Use of the guidance in the manuscript** |
| --- | --- | --- | --- |
| **The Methodology**  NB: We prefer the theoretically-embedded term Methodology as a section header, over the proceduralist term Method. | | | |
| ***Participants/data items*** | | | |
| Describe selection of participants/data items. | Should include criteria for selection and/or recruitment strategies and settings. | Terms “sample/sampling”, which connote “sampling” from a population (for the purpose of statistical generalisation). | We describe how participants were identified and recruited, and the approach to this. We outline a ‘maximum variation’ approach but avoid the use of the term sampling. |
| Describe number of participants/data items; provide a rationale or explanation around dataset or participant group size/composition. | Non-positivist qualitative concepts, such as “information power” or sufficiency offer conceptually appropriate justifications for “dataset” or “participant group” size and composition.8 | Justification based on saturation (simple realist), or statistical models (positivist); reporting rates of non-participation (an indicator of the representativeness of the “sample” in quantitative research). | We describe the number of participants and the justification of ensuring an equal representation of participant groups in the total composition. |
| Discuss characteristics of participants/data items. | Balance the need to “situate the participant group” with participant anonymity (e.g., aggregate or report minimal demographics where appropriate).^6^ | Tables with each participant’s demographic information listed line-by-line. | We describe participant characteristics through aggregated reporting. |
| Detail ethical approval and ethical code/principles followed, participant informed consent, etc. | Ethical discussion usually includes institutional ethical approval (if needed), but may include wider principles; providing research materials (participant information, consent form, etc.) in supplementary materials may be useful to support reflexive openness. | Compromising participant anonymity by the details provided. | Ethical approval is detailed, as are informed consent procedures. |

| **Advice for aspects of the research report/approach to reporting** | **Guiding notes and further explanation** | **Practices, concepts and terminology to avoid** | **Use of the guidance in the manuscript** |
| --- | --- | --- | --- |
| **The Methodology (Continued)**  NB: We prefer the theoretically-embedded term Methodology as a section header, over the proceduralist term Method. | | | |
| ***Dataset generation***  *NB: We prefer the term generation over collection to capture the active role of the researcher and that data don’t pre-exist research as data, but become data through research practices.* | | | |
| Provide some rationale for method(s) for data generation/data item sources chosen. | Discuss why the method(s) of data generation/data source was a good fit with the research question, participant group, guiding theory, etc. If multiple data sources are used, any rationale for combination should be conceptually appropriate (e.g., crystallisation^9^). | Triangulation as a rationale for different data sources (realist). | We outline in particular the choice of transition from workshops to focus groups as being led through feedback from participants as more conducive to genuine engagement. |
| Describe development and/or characteristics of data generation tool(s). | Include tool(s) in supplementary materials when possible; discuss piloting if used, and any changes following piloting, or during data generation. | Using an existing tool with the aim of replicating existing “findings”, or developing and describing a tool in a way that is intended to facilitate future replication (positivist). | We provide the guides for all workshops and focus groups, as well as surveys, as part of the supplementary materials. We specifically detail how development of workshops and focus groups were informed by McKercher’s (2020) theoretical concepts, as well as other sources (e.g., Metro North Health (2022)). |
| Include details such as modality and/or setting of data generation, time frame, and other pertinent procedural information. | Relevant information includes: the mode of a data generation tool (e.g., video call focus groups; chat-based interviews); the context of data generation (location; timeframe) – where this doesn’t compromise participant anonymity; and mode of recording interactive data generation. | Standardisation as a gold standard (realist); justifying an aimed for standardisation in data generation tools as a means to facilitate the “reliability” or “accuracy” of the research; treating a lack of standardisation in data generation method, modality or setting as a problem, a potential source of “bias”. | We provide in-depth detail about modality, setting and timeframes – incorporating further contextual information (e.g., Co-facilitators meeting participants at a familiar location outside a nearby mental health centre and walked together to the university for those who felt more comfortable with this.). |
| Describe who conducted any interactive data generation (which author or research role), and how. | Can include what, if anything, the researcher disclosed about their personal or professional positioning or motivation; what skills and experience they brought; note researcher’s relationship with participants prior to, during and after the research. | Seeking standardisation (e.g., through the training of researchers) in interactive data collection; treating non-standardisation as a threat to “reliability” or “accuracy”. | We explicitly identify the researchers involved in interactive data generation, as well as their positionality and roles through Box 2. We also describe the embedded researchers’ relationships with participants and how this may have influenced the co-design process. |
| Describe the size/scope of dataset and dataset items. | Such as the range and average length for interviews/focus groups; range and average word length for textual data items. | Equating data quantity with data quality. | We describe the length of workshops, focus groups and structured power reflection. |
| Describe, and if relevant explain, any preparation of data for analysis. | Such as method of transcription of audio/video data (a transcription key can go in supplementary materials); changes and “corrections” – such as why typographical errors in written data were corrected; system for removing any identifying information; use of pseudonyms and/or data codes. | Describing transcription as “verbatim” or “orthographic” with no further details; using edited or “cleaned up” data without acknowledgement of this; participant validation of the “accuracy” of transcripts (realist). | We describe how data were transcribed, addressing typographical errors and removal of identifying information. |

| **Advice for aspects of the research report/approach to reporting** | **Guiding notes and further explanation** | **Practices, concepts and terminology to avoid** | **Use of the guidance in the manuscript** |
| --- | --- | --- | --- |
| **The Methodology (Continued)**  NB: We prefer the theoretically-embedded term Methodology as a section header, over the proceduralist term Method. | | | |
| ***Data analysis*** | | | |
| Provide some rationale for use of RTA, and, where relevant, for combining RTA with other approaches and procedures. | Any combining of RTA with other method/ologies or procedures should be warranted, rather than based on a misunderstanding of RTA, and conceptually coherent (unless clearly justified). | Citing generic characteristics of RTA (e.g., accessible, flexible) without explaining how they were relevant to the study; using a codebook without acknowledging this is not part of RTA and justifying its use. | We describe the rationale for the use of RTA as being consistent with the co-design methodology. |
| Describe specific orientation to RTA. | Locate RTA on dimensions of inductive<>deductive and semantic<>latent. | A generic discussion of TA (or even RTA), not specifically situated in relation to the study or approach. | We locate our RTA as inductive and both semantic and latent during initial stages, as a tool to guide reflexivity in discussion about meaning imposed and constructed by each researcher. |
| Discuss how the researcher(s) engaged with the analytic process. | Provide a specific and situated account of the analysis process; use supplementary materials to provide a fuller account of the analytic process. | Offering a generic description of the six phases of RTA in lieu of an account of analytic process. | We provide a rich account of how analysis was carried out. |
| Where more than one person is involved, describe who analysed the data (author or research role). | Role(s) or involvement throughout the process should be discussed; where coding was collaborative, what this involved and how differences in coding and theme development were tackled, should be included. | Use of inter-coder agreement measures, consensus coding approach (positivist). | We identify how coding was collaborative and how divergence was addressed. |
| Use language to describe the process and products of RTA that is coherent with the values and assumptions of RTA. | Language should convey the active role of the researcher(s) in “generating”, “crafting”, “constructing”, “creating”, “producing” or “developing” themes; language around themes should evokes them as products of a researcher-data process. | Passive language of discovery, such as “emerging”, “found”, “identified”, “discovered” – these evoke themes as “diamonds scattered in the sand” (p. 740)10; unexplained use of language and concepts from other approaches, such as emergent or superordinate themes (IPA), or line-by-line and/or open coding and constant comparison (grounded theory). | We have attempted to convey the active role of the researchers consistently throughout the paper. |

| **Advice for aspects of the research report/approach to reporting** | **Guiding notes and further explanation** | **Practices, concepts and terminology to avoid** | **Use of the guidance in the manuscript** |
| --- | --- | --- | --- |
| **The Analysis**  NB: We prefer the heading Analysis over Findings/Results. Findings implies the researcher “found”, “discovered” or “identified” pre-existing themes. Results is strongly associated with the outputs of statistical analysis. | | | We have used the heading ‘Results’ as this section combines both qualitative and quantitative analysis. |
| ***Reporting the data analysis*** | | | |
| Provide an overview of themes or thematic structure. | Overviews can include a list, map or table of themes to preview the analysis. | An unclear thematic structure, including unexplained headings in the Analysis. | We provide an overview of analytic categories and interpretative themes through Figure 1 and in-text. |
| Ensure theme conceptualisation is appropriate to RTA, and any divergences are justified and explained. | In RTA, themes report shared meaning, united around a central organising concept that differs for each theme. | Topic summaries; data generation questions reported as “themes”. | We discuss shared meaning around a central concept (e.g., challenges relating to power dynamics, duration and timing of sessions, and the use of technical language). |
| Name themes appropriately. | Use theme names that capture the “essence” or “story” of each themes; brief data quotations can be used. | (One-word) theme names that only identify a topic, and offer no story (evoking topic summaries). | We have used one-word theme names sparingly, where there is often greater plurality of shared meaning within the theme. |
| Report themes in sufficient depth and detail. | As RTA is an interpretative method, themes should be multifaceted, and contain both data and analytic narrative; if useful, additional data extracts may be included in supplementary materials.^7^ | Thin, one dimensional themes, effectively conflating codes and themes; large number of themes relative to the length of the manuscript. | We have provided multiple data for each theme and sub-theme, accompanied by analytic narrative. |
| Use subtheme judiciously. | Themes are the main analytic purpose, and should be multifaceted; only use subthemes where doing so highlights an important facet or aspect of the central concept of a theme. | Fragmenting the analysis through overuse of subthemes, and an overly elaborated/“bitty” thematic structure. | We have used sub-themes sparingly (twice). |
| Ensure the analytic narrative explains the meaning and significance of the data. | For RTA, each theme needs an analytic narrative that outlines its meaning and importance in relation to the topic, research question and dataset; the reader needs to be told about why/how data excerpts matter and “evidence” the theme; the Analysis section also needs to convey the overall story of the analysis. | Frequency counts as a justification for themes presented; simple paraphrasing of data as “analytic narrative”; treating data meaning as self-evident (data are assumed to speak for themselves); “arguing” with the data (treating the data as something to [dis]agree with, rather than to interpret and make sense of). | We have attempted to provide an analytic narrative for all data, contextualizing this and describing explicitly our interpretation. |
| Provide an appropriate balance of analytic narrative and data extracts – both data extracts and analytic narrative matter. | The rich descriptive and/or interpretative story of the analysis needs to be woven around sufficient analytic extracts from across the dataset. | Presenting either a long string of data extracts with barely any analytic narrative, or only the researcher’s narrative summary of the theme, without any data extracts to support it. | We have attempted to strike this balance. |
| Demonstrate coherence between analytic narrative and illustrative/evidentiary data extracts. | Data extracts should convincingly and compellingly evidence the analytic claims. | Mismatches between data extracts and analytic claims; not countering obvious alternative readings of the data. | Where language is idiosyncratic or highly contextualized we have tried to unpack this to appropriately link it with the analytic claims. |
| Integrate existing research and theory into the analytic narrative. | In RTA, an interpretative analytic narrative is enriched by incorporating relevant existing research and theory into the reporting of themes, reflecting notions of contextualised meaning, and contributing to an ongoing “conversation” about a topic. | The positivist tradition of separating a description of analytic “Results” and their interpretation with reference to scholarship and theory in a “Discussion” section. | We have minimally integrated existing research in this section. |

| **Advice for aspects of the research report/approach to reporting** | **Guiding notes and further explanation** | **Practices, concepts and terminology to avoid** | **Use of the guidance in the manuscript** |
| --- | --- | --- | --- |
| **The Final Section – A General Discussion or “Conclusions”**  NB: We don’t have a preference for what a final section of an RTA report is called, and it depends on the context and the focus and purpose of the study – the heading Conclusion may evoke a certainty that isn’t appropriate; Implications may be useful; Final Considerations or Reflections may work, as might General Discussion. | | | |
| ***Quality, evaluation and conclusions*** | | | |
| Draw analytic conclusions across themes. | Orient to the “so what” of the overall analysis – the “point” of the story told; this might include discussion of implications for practice and “actionable” outcomes.^11^ | Repetitive theme-by-theme integration of the analysis with existing literature; no overall conclusions drawn; no overall analytic story. | We have attempted to highlight actionable outcomes as practical tips and draw conclusions across themes. |
| Discuss implications or directions for future research. | Any suggestions for future research should stem from the analysis and be evidence-based (e.g., provide grounds for other groups potentially having different experiences or views) rather than generic. | Generic recommendations for other research, such as with a different “population”. | Our suggestions for future research are methodologically-based, rather than focusing on replication in different populations. |
| Use and report quality practices coherent with RTA. | Ensure evaluation of research quality deploys conceptually coherent notions, such as: member reflections; crystallisation;^9^ others serving as a critical friend/sounding board to enhance insight;^12^ reflexive journaling. | Incoherent quality measures such as: member checking/participant validation; triangulation (realist); the use of theme agreement/consensus among researchers or corroboration of themes by another researcher (positivist). | We use reflexivity practices coherent with RTA, such as field notes and crystallisation. |
| Evaluate the research from a Big Q standpoint. | Such evaluation might including considering how the specifics of the study may have shaped the research produced (for example, the characteristics and context of the participant group/dataset; the methods and modalities for generating the data); situatedness should not be treated as a limitation. | Evaluations and descriptions of limitations that orient to quantitative or positivist norms, such as reference to lack of generalisability – positioned as a limitation, and equated only with statistical generalisability13 – or a “small” (by implication non-ideal) and “unrepresentative” “sample”. | Through our power reflection we explicitly interrogative our study from a Big Q perspective. |
| Include reflections on research process and practices, including researcher reflexivity. | Some consideration of the researcher(s)’s role in shaping the research and the knowledge generated is an important quality marker. | Reference to researcher bias/influence (positivist). | We include reflections on roles in shaping knowledge through the description of the embedded researchers and the structured power reflection. |

# References

1. Braun V and Clarke V. One size fits all? What counts as quality practice in (reflexive) thematic analysis? *Qual. Res. Psychol*. 2021; 18: 328-352.
2. Braun V and Clarke V. Conceptual and design thinking for thematic analysis. *Qual Psychol.*

2022; 9: 3-26.

3. Braun V and Clarke V. *Thematic analysis: A practical guide*. London: Sage, 2022.
4. Braun V and Clarke V. How do we solve a problem like COREQ? A critique of Tong et al.’s (2007) consolidated criteria for reporting qualitative research. Unpublished.
5. Braun V and Clarke V. Reporting guidelines for qualitative research: A values-based approach. Unpublished.
6. Elliot R, Fischer CT and Rennie DL. Evolving guidelines for publication of qualitative research studies in psychology and related fields. *Br. J. Clin. Psychol.* 1999; 38: 215-229.
7. Levitt HM, Motulsky SL, Wertz FJ, Morrow SL and Ponterotto JG. Recommendations for designing and reviewing qualitative research in psychology: Promoting methodological integrity. *Qual Psychol.* 2017; 4: 2-22.
8. Malterud K, Siersma VK and Guassora AD. Sample size in qualitative interview studies: Guided by information power. *Qual. Health Res.* 2016; 26: 1753-1760.
9. Tracy SJ. Qualitative quality: Eight “big-tent” criteria for excellent qualitative research. *Qual Inq.* 2010; 16: 837-851.
10. Braun V and Clarke V. (Mis)conceptualising themes, thematic analysis, and other problems with Fugard and Potts’ (2015) sample-size tool for thematic analysis. *Int. J. Soc. Res. Methodol*. 2016; 19: 739-743.
11. Sandelowski M and Leeman J. Writing usable qualitative health research findings.

*Qual. Health Res.* 2012; 22: 1404-1413.

12. Smith B and McGannon KR. Developing rigour in qualitative research: Problems and opportunities within sport and exercise psychology. *Int Rev Sport Exerc Psychol.* 2017; 11: 101-121.
13. Smith B. Generalizability in qualitative research: Misunderstandings, opportunities and recommendations for the sport and exercise sciences. *Qual. Res. Sport Exerc. Health.* 2018; 10: 137-149.
