# Supplementary material for: ‘That Essence of Empowerment’: An Exploration of People Living With Mental Illness', Healthcare Professionals', and Researchers' Experiences of Co‐Design
Source: Health Expect. 2026 Oct 15;29(5):e70888. doi: 10.1111/hex.70888 (PMC13670414; doi:10.1111/hex.70888)
Supplement: Supplementary file 4 [file HEX-29-e70888-s004.docx]

# Supplementary File 2 – GRIPP2-SF

| **Section and topic** | **Item** |
| --- | --- |
| 1: Aim   Report the aim of the study | This study aimed to: (a) describe in detail the co-design process undertaken (‘How was co-design enacted?’), (b) understand People with Lived/Living Experience’s PWLE), Health Care Professionals’ (HCPs), and researchers’ experiences of participating in the same co-design process (‘How was co-design experienced?’), and (c) critically reflect on the power dynamics throughout the co-design process (‘How was power manifest in the co-design?’). |
| 2: Methods   Provide a clear description of the methods used for PPI in the study | PPI contributed to the study in several ways, including:  - Funding acquisition (Lived/Living Experience (LE) co-lead)  - Conceptualisation of the study and methodological design (LE co-lead)  - Co-facilitation of the workshops and focus groups (LE co-lead and peer worker)  - Conducting qualitative analysis (LE co-lead & LE co-researcher)  - Collaboratively writing the first draft of the manuscript (LE co-lead)  - Reviewing the manuscript (LE co-researcher)  - Ensuring community-grounded dissemination pathways (e.g., through the mental health service and Consumer Advisory Groups) (LE & HCP representation on the Steering Committee)  - Reviewing ethics applications and guides for focus groups (LE & HCP representation on the Steering Committee)  - Developing participant feedback summaries (LE co-lead (workshops); LE co-lead and LE & HCP representation on the Steering Committee (focus groups)) |
| 3: Results   Outcomes—Report the results of PPI in the study, including both positive and negative outcomes | This involvement led to several outcomes, including:  - Ensuring a more diverse audience and reach of the research results through the additional dissemination pathways  - Ensuring that the methodology was adapted to increase engagement with PWLE participants (from workshops to focus groups)  - Creating more effective and accessible feedback loops, through emphasising the need to ask each participant about learning styles, reframing technical language from summaries, ensuring deadlines for feedback loops were met  - Making the manuscript language more accessible to a non-academic audience  - Increasing reflexivity throughout the analysis process  - Creating and safe and inclusive environment in co-design sessions |
| 4: Discussion   Outcomes—Comment on the extent to which PPI influenced the study overall. Describe positive and negative effects. | LE involvement in this study was very effective and influenced important aspects of the study, based on the impacts in Section 3. This might have been related to several factors. Firstly, the LE co-lead is highly experienced in co-design methodologies. They also received ongoing training and supervision in conducting qualitative analysis and academic writing from the conventional researchers on the team. In addition, the LE co-researcher is highly experienced with qualitative research.  The right processes were in place, as the LE co-lead was involved from the beginning of the study allowing them to help shape the study fully. Having the right context, with a collaborative research team, funding to finance their time, and a supportive attitude of their involvement also likely assisted in the positive impact that PPI had on this study. Pre-existing relationships between the embedded researchers and PWLE and HCP participants also probably benefitted the degree of engagement through recruitment. |
| 5: Reflections   Critical perspective—Comment critically on the study, reflecting on the things that went well and those that did not, so others can learn from this experience | LE in the study was deeply enmeshed through being co-led by a LE researcher and enhanced through the formation of the Steering Committee. The aim of active collaboration in an attempt to co-produce knowledge worked well. The key challenge was the timescale required to ensure genuine reflexivity and collaboration throughout the writing process, and for learning new skills for the LE co-lead. We are aware that this process might have limited engagement with the research due to extended timelines. Other challenges, including managing expectations and power dynamics, are discussed within the study in the Results section. |
