# Supplementary material for: ‘That Essence of Empowerment’: An Exploration of People Living With Mental Illness', Healthcare Professionals', and Researchers' Experiences of Co‐Design
Source: Health Expect. 2026 Oct 15;29(5):e70888. doi: 10.1111/hex.70888 (PMC13670414; doi:10.1111/hex.70888)
Supplement: Supplementary file 5 [file HEX-29-e70888-s005.docx]

**Focus Group Guide (Co-facilitators-only)**

- Did we achieve a positive change or make things better? If so, how was that reflected?
- Did we use a collaborative co-design process to work together? If so, what was the benefits of this process?
  - What worked well during the co-design process?
  - What aspects do you think didn’t work so well?
- Were there any stages of the co-design process that you found challenging? If so, what were these and why?
  - What steps were taken to overcome these challenges?
- What stages stood out as successful and why?
- Did you feel that the co-design process achieved a positive outcome? If so, what and why?
- Did you feel that your skills and confidence increase around co-design in the future? If so, how and why?
- Do you feel you benefited from participating in the co-design? If so, how and why?
- Have relationships been built within the team that will continue after the project?
