# Supplementary material for: ‘That Essence of Empowerment’: An Exploration of People Living With Mental Illness', Healthcare Professionals', and Researchers' Experiences of Co‐Design
Source: Health Expect. 2026 Oct 15;29(5):e70888. doi: 10.1111/hex.70888 (PMC13670414; doi:10.1111/hex.70888)
Supplement: Supplementary file 6 [file HEX-29-e70888-s006.docx]

**Part 1**

1. What was your date of birth?
2. How do you describe your gender?

☐	Man or male

☐	Woman or female

☐	Non-binary

☐	I use a different term (please specify) __________________________

☐	Prefer not to answer

3. How would you describe your cultural background? Your cultural background is the cultural/ethnic group(s) to which you feel you belong or identify. This background may be the same as your parents, grandparents, or your heritage, or it may be the country you were born in or have spent a great amount of time in, or you feel more closely tied to.
4. Which language do you mainly speak at home?

☐	English (skip question 5)

☐	Other

5. How well do you speak English?

☐	Very well

☐	Well

☐	Not well

☐	Not at all

6. Are you of Aboriginal or Torres Strait Islander origin? (For persons of both Aboriginal and Torres Strait Islander origin, tick both 'Yes' boxes.)

☐	No

☐	Yes, Aboriginal.

☐	Yes, Torres Strait Islander.

7. Which of the following best describes your employment status?

☐	Full-time employee

☐	Part-time employee

☐	Unemployed

8. Which of the following best describes the accommodation you live in?

☐	Own home (owned or partly owned by me)

☐	Renting

☐	Board and lodgings

☐	Government rent

☐	No rent paid

9. How long have you been connected with mental health services?

_________ years	(&)	________ months

**Part 2**

10. Have you participated in a co-design workshop in the past?

☐	Yes

☐	No

11. Did your understanding of co-design improve from attending this workshop?

☐	Yes

☐	No (Please provide comment)

Comments:

12. Did you feel that you received sufficient information prior to the workshop to understand the purpose?

☐	Yes

☐	No (Please provide comment)

Comments:

13. What did you like most about the workshop?

Comments:

14. What do you like least about the workshop?

Comments:

15. How likely are you to take part in future co-design workshops?

☐	Very Likely

☐	Somewhat Likely

☐	Not at All (Please provide comment)

Comments

16. I felt that my perspective was valued in this co-design process as much as others.

☐	Strongly agree

☐	Somewhat agree

☐	Neutral

☐	Somewhat disagree

☐	Strongly disagree

17. I felt supported to participate in this co-design process.

☐	Strongly agree

☐	Somewhat agree

☐	Neutral

☐	Somewhat disagree

☐	Strongly disagree

18. I felt that my input has been accurately reflected in the feedback.

☐	Strongly agree

☐	Somewhat agree

☐	Neutral

☐	Somewhat disagree

☐	Strongly disagree

19. How confident are you that the outcomes of this process will result in an improved service?

☐	Confident

☐	Neutral

☐	Not confident

20. Please rate your overall experience.

☐	Excellent

☐	Good

☐	Poor

21. Is there anything else you would like to add that could help improve this area of the service?

Comments:
