# Supplementary material for: ‘That Essence of Empowerment’: An Exploration of People Living With Mental Illness', Healthcare Professionals', and Researchers' Experiences of Co‐Design
Source: Health Expect. 2026 Oct 15;29(5):e70888. doi: 10.1111/hex.70888 (PMC13670414; doi:10.1111/hex.70888)
Supplement: Supplementary file 7 [file HEX-29-e70888-s007.docx]

**Part 1**

1. What was your date of birth?
2. How do you describe your gender?

☐	Man or male

☐	Woman or female

☐	Non-binary

☐	I use a different term (please specify) __________________________

☐	Prefer not to answer

3. Which of the following best describes your profession?

☐	Psychiatrist ☐ Occupational Therapist

☐	Psychologist	☐ Social Worker

☐	Nurse	☐ Physiotherapist

☐	Dietitian	☐ Other ___________________

☐	Exercise Physiologist

4. How long have you worked in mental health services?

_________ years	(&)	________ months

**Part 2**

5. Have you participated in a co-design workshop in the past?

☐	Yes

☐	No

6. Did your understanding of co-design improve from attending this workshop?

☐	Yes

☐	No (Please provide comment)

Comments:

7. Did you feel that you received sufficient information prior to the workshop to understand the purpose?

☐	Yes

☐	No (Please provide comment)

Comments:

8. What did you like most about the workshop?

Comments:

9. What do you like least about the workshop?

Comments:

10. How likely are you to take part in future co-design workshops?

☐	Very Likely

☐	Somewhat Likely

☐	Not at All (Please provide comment)

Comments

11. I felt that my perspective was valued in this co-design process as much as others.

☐	Strongly agree

☐	Somewhat agree

☐	Neutral

☐	Somewhat disagree

☐	Strongly disagree

12. I felt supported to participate in this co-design process.

☐	Strongly agree

☐	Somewhat agree

☐	Neutral

☐	Somewhat disagree

☐	Strongly disagree

13. I felt that my input has been accurately reflected in the feedback.

☐	Strongly agree

☐	Somewhat agree

☐	Neutral

☐	Somewhat disagree

☐	Strongly disagree

14. How confident are you that the outcomes of this process will result in an improved service?

☐	Confident

☐	Neutral

☐	Not confident

15. Please rate your overall experience.

☐	Excellent

☐	Good

☐	Poor

16. Is there anything else you would like to add that could help improve this area of the service?

Comments:
