# Supplementary material for: ‘That Essence of Empowerment’: An Exploration of People Living With Mental Illness', Healthcare Professionals', and Researchers' Experiences of Co‐Design
Source: Health Expect. 2026 Oct 15;29(5):e70888. doi: 10.1111/hex.70888 (PMC13670414; doi:10.1111/hex.70888)
Supplement: Supplementary file 8 [file HEX-29-e70888-s008.docx]

| Timing | Section | Facilitator | Activity | Tasks | Resources |
| --- | --- | --- | --- | --- | --- |
| 10:00 – 10:15  (15 mins) | Welcome |  | - **Acknowledgement of country and lived experience.** - **Intro Facilitation Team** - **Agenda and Breaks** - Support available - Something you want to get out of today - Recap & relay to next section | Presentation | Slide 1: Welcome  Slide 2: Agenda |
| 10:30 – 1100  (30 mins) | Reflection |  | - Reflection on working together (co-design) | Presentation | Slide 3: What is co-design  Slide 4: Architecture |
| 10.15 – 10.30 (15 mins) | Introduction to workshop 2. |  | - What did we do in Workshop 1 & what did we find out? - Why are we here for Workshop 2? - Transparency for ideas from Workshop 1. | Presentation. | Slide 5: Workshop 1  Slide 6: Why are we here?  Slide 7: Ideas from workshop 1  Slide 8 – 12: Potential solutions |
|  |  |  | - Priority setting potential solutions:   - Peer-supported upskilling program   - Mobile phone application   - Healthy food incentive program   - Food bank / pantry   - Meal kit & frozen meal delivery | Participants rank listed solutions in order of priority. | Slide 13: Prioritising potential solutions.  Hardcopy list of solutions. |
|  | | | | | Slide 14: Coffee break |
| 11:15 – 11.30  (15 mins) | Models of care |  | - Feedback on priorities - Understanding a model of service: who (it for and who does it), what, where, when, how, anything else (WWWWHA). - Example: youth Brief Intervention Service | Presentation | Slide 15: Priorities  Slide 16: Developing a Service Model  Slide 17 & 18: Example |
| 11:30 – 12:30  (60 mins) | Solution 1 |  | - Intro to activity (developing models of care) - Intro to solution 1 - Small group brainstorming - Reporting back to group and whole group discussion. - Intro AIM IAM FIM questionnaire. - Assess feasibility and acceptability. | Small group – WWWH on butcher’s paper.  Whole group discussion.  Give AIM IAM FIM form to participants. | Slide 19: Solution 1  AIM IAM FIM Measure |
|  | | | | | Slide 20: Lunch break |
| 1:15 – 2:15  (60 mins) | Solution 2 |  | - Intro to solution 2 - Small group brainstorming - Reporting back to group and whole group discussion. - Assess feasibility and acceptability. | Small group – WWWH on butcher’s paper.  Whole group discussion.  Give AIM IAM FIM form to participants. | Slide 21: Solution 2  AIM IAM FIM Measure |
|  | | | | | Slide 22: Coffee break |
| 2:30 – 3:30  (60 mins) | Solution 3 |  | - Intro to solution 3 - Small group brainstorming - Reporting back to group and whole group discussion. - Assess feasibility and acceptability. | Small group – WWWH on butcher’s paper.  Whole group discussion.  Give AIM IAM FIM form to participants. | Slide 23: Solution 3  AIM IAM FIM Measure |
|  | | | | | Slide 24: Stretch |
| 3.35 – 3.50  (15 mins) | Solution reflection |  | - Reflection on potential solutions - Reflect on AIM IAM FIM scores. - Which seems most feasible & acceptable? - Have people changed their priorities? - Grouping of solutions activity | Tally scores.  Each table to group solutions (if desired) to form a priority package of care. | Slide 25: Reflection on potential solutions  Cards with each solution for grouping activity. |
| 3:50 ­– 4:00  (10 mins) | Evaluation & Close |  | - Your Experience Survey - Around the room: one word to describe how you are feeling, or your experience of today or something you will do for yourself afterwards | Hand out survey to participants | Slide 26: Evaluation  Slide 27: Thank you  Survey Handout |
