# Supplementary material for: ‘That Essence of Empowerment’: An Exploration of People Living With Mental Illness', Healthcare Professionals', and Researchers' Experiences of Co‐Design
Source: Health Expect. 2026 Oct 15;29(5):e70888. doi: 10.1111/hex.70888 (PMC13670414; doi:10.1111/hex.70888)
Supplement: Supplementary file 9 [file HEX-29-e70888-s009.docx]

# Power Literacy – v2

- Power is not inherently positive or negative. Power is *choice and control*.
- *Power to*: Power is a something—anything—which makes or renders somebody able to do, capable of doing something. Power is capacity, potential, ability, or wherewithal.
- *Power over*: The relational capacity that enables a social actor to influence asymmetrically the decisions of other social actor(s) (i.e., getting someone to do what you want them to do).
- *Power with*: The power is in the room; you can choose to use it or not as you may see fit.

| **Domain** | **Definition** |
| --- | --- |
| *Privilege* | Influence due to an unearned advantage based on social position or identity (e.g., white, male, able-bodied, straight). |
| *Access* | The ability to influence who is included and who is excluded from a  service design project. |
| *Goal* | The ability of designers to initiate and frame the design project, including the way in which problems are defined, goals are chosen, and the design project is structured. |
| *Role* | The ability to influence the roles that different actors—  those who have already been given access to the project—will  assume during a design project. |
| *Rule* | The ability to establish the way that actors included in the design network will work together. This often pertains to what designers consider ‘rational’, ‘normal’, or ‘valid’. |

- Let’s reflect consistently on all three groups: people with lived experience, healthcare professionals, and researchers.

| **Domain** | **Reflection** |
| --- | --- |
| *Privilege* | - What privilege do you have and how does it differ from those you are working with? - How might this have affected relationships with the community that we were designing with? - Who may have been unintentionally excluded or marginalised as a result? |
| *Access* | - Who was included and excluded in the design project? For what reasons? - How were different stakeholders invited to participate? - Was the quality of participation the same throughout the design process, or did it change? |
| *Goal* | - Who initiated this project and what problems, desired outcomes, and processes had already been decided/what were open to change? - How might framing and goals for this project have affected participation? |
| *Role* | - What different roles were given to stakeholders (e.g., participant, team member, expert, researcher, decision maker, nonparticipant) and who decided on this? - Which actors had the ability to interpret and/or prioritise findings? - What effect, good or bad, might these roles have had on stakeholders with lived experience outside of the design project? |
| *Rule* | - How might rules, norms, and beliefs guiding the way we worked together and made decisions have amplified or silenced voices? - What effects might this have had for this project and more broadly for the community we were working with? |
