# Supplementary material for: ‘That Essence of Empowerment’: An Exploration of People Living With Mental Illness', Healthcare Professionals', and Researchers' Experiences of Co‐Design
Source: Health Expect. 2026 Oct 15;29(5):e70888. doi: 10.1111/hex.70888 (PMC13670414; doi:10.1111/hex.70888)
Supplement: Supplementary file 10 [file HEX-29-e70888-s010.docx]

**Focus Group Guide (PWLE & HCP; PWLE-only)**

*CFIR Questions*

- How do you feel about the intervention being used in your setting (e.g., community mental health centre)?
  - Why?

**15 MINUTE MARK**

- How does the intervention compare to other alternatives that may have been considered or that you know about?
  - For example, OzHarvest, soup kitchens, food pantries.
- How does the intervention compare to other similar existing programs in your setting?
  - For example, Keeping the Body in Mind?

**30 MINUTE MARK**

- What barriers will people face to participating in the intervention?
- How well does the intervention fit with existing practices in your setting?
- What kinds of changes or alterations do you think you will need to make to the intervention so it will work effectively in your setting?

**45 MINUTE MARK**

- What kinds of infrastructure changes will be needed to accommodate the intervention?
- How well do you think the intervention will meet the needs of the individuals served by your organisation?
- What kind of information should we also be collecting to determine if the intervention is effective?

**65 MINUTE MARK**

*Co-Design Questions*

- Could you describe what worked well during the co-design process?
- What aspects do you think didn’t work so well?
- Were there any challenges?
  - If so, what were they and what steps were taken to overcome these challenges?

**80 MINUTE MARK**

- Do you think everyone was engaged in the workshops, and did this change over time?
  - If not, what do you think might have prevented people from engaging with the workshops?
- Do you feel you benefited from participating in the workshops? If so, how?
- Is there anything else you would like to add about your experience that we haven’t asked about?

**90 MINUTE MARK**
